# Plant Disease Detection and Classification: A Systematic Literature Review

**DOI:** 10.3390/s23104769

**Published:** 2023-05-15

**Authors:** Ramanjot, Usha Mittal, Ankita Wadhawan, Jimmy Singla, N.Z Jhanjhi, Rania M. Ghoniem, Sayan Kumar Ray, Abdelzahir Abdelmaboud

**Affiliations:** 1 Department of Computer Science and Engineering, Lovely Professional University, Phagwara 144411, Punjab, India; 2 School of Engineering and Technology, CT University, Ludhiana 142024, Punjab, India; 3 School of Computer Science, SCS, Taylor’s University, Subang Jaya 47500, Malaysia; 4 Department of Information Technology, College of Computer and Information Sciences, Princess Nourah bint Abdulrahman University, P.O. Box 84428, Riyadh 11671, Riyadh, Saudi Arabia; 5 Department of Information Systems, King Khalid University, Abha 61913, Muhayel Aseer, Saudi Arabia

**Keywords:** convolutional neural network, machine learning, deep learning, image processing, disease identification

## Abstract

A significant majority of the population in India makes their living through agriculture. Different illnesses that develop due to changing weather patterns and are caused by pathogenic organisms impact the yields of diverse plant species. The present article analyzed some of the existing techniques in terms of data sources, pre-processing techniques, feature extraction techniques, data augmentation techniques, models utilized for detecting and classifying diseases that affect the plant, how the quality of images was enhanced, how overfitting of the model was reduced, and accuracy. The research papers for this study were selected using various keywords from peer-reviewed publications from various databases published between 2010 and 2022. A total of 182 papers were identified and reviewed for their direct relevance to plant disease detection and classification, of which 75 papers were selected for this review after exclusion based on the title, abstract, conclusion, and full text. Researchers will find this work to be a useful resource in recognizing the potential of various existing techniques through data-driven approaches while identifying plant diseases by enhancing system performance and accuracy.

## 1. Introduction

Agricultural biodiversity is foundational to providing food and raw materials to humans. When pathogenic organisms such as fungi, bacteria, and nematodes; the soil pH; temperature extremes; changes in the amount of moisture and humidity in the air; and other factors continuously disrupt a plant, it can develop a disease. Various plant diseases can impact the growth, function, and structures of plants and crops, which automatically affect the people who are dependent on them. The majority of farmers still use manual methods to identify plant illnesses, since it is challenging to do so early on and has a negative impact on productivity. To overcome this, many deep learning (DL), image processing, and machine learning (ML) techniques are being developed, by which the detection of disease in a plant is performed by images of plant leaves.

Image processing is utilized to improve the quality of images in order to extract valuable information from them; because of this feature, image processing techniques are utilized in many areas, such as color processing, remote sensing, and pattern recognition, of the medical and agricultural fields. Images of plant leaves can be used to identify disease using image processing techniques that are appropriate, effective, and dependable. In image processing techniques, various stages are involved—image acquisition, image pre-processing, feature extraction, image segmentation, and classification. In this study, we examined papers that use image processing approaches. For an instance, Malathy et al. [1] claim that 97% classification accuracy can be achieved for illness detection using image processing techniques, which is highest compared to other publications.

Machine learning (ML) involves the concept of loss function, which makes it more effective than image processing. The loss function lets us know how the proposed models will function via predictions. Models can predict outcomes more correctly when machine learning (ML), a type of artificial intelligence (AI), is used without explicit guidance. Models which are trained using ML improve their performance based on experience. Due to its nature, ML is applicable in many fields, some of which are medical diagnosis, speech recognition, product recommendation, self-driving cars, virtual assistants (such as Alexa and Siri), prediction of traffic (for example—in Google Maps), and agriculture. ML approaches can be implemented in various manners to develop new algorithms for detecting and classifying diseases that occur in plants at an early stage. We reviewed the state-of-the-art literature in this field, and found that Rumpf et al. [2], Dubey et al. [3], Ramesh et al. [4], Behera et al. [5], Tulshan et al. [6], Wahab et al. [7], etc., are utilizing the concept of ML.

Deep learning (DL) networks learn by spotting intricate patterns in the data with which they work. By building computational models that are composed of numerous processing layers, the networks can produce various degrees of abstraction to explain the data. Although DL is a form of ML, it is more adaptable than ML. While feature extraction and classification are carried out separately in ML, they are combined by utilizing numerous processing layers in DL. When working with unstructured data, automatic feature generation, superior self-learning capabilities, and support for distributed and parallel algorithms are all areas in which it outperforms ML. Various DL approaches can be utilized in agriculture for detecting diseases in plants from leaves, such as recurrent neural networks (RNNs) and convolutional neural networks (CNNs). The proposed paper reviewed the state-of-the-art works by Sladojevic et al. [8], Fujita et al. [9], Brahimi et al. [10], Fuentes et al. [11], Cap et al. [12], Ma et al. [13], Sardogan et al. [14], Adedoja et al. [15], Geetharamani et al. [16], Zhang et al. [17], Sharma et al. [18], Coulibaly et al. [19], Ji et al. [20], Marzougui et al. [21], Shrestha et al. [22], Selvam et al. [23], Jadhav et al. [24], Lijo [25], Sun et al. [26], Sujatha et al. [27], Abbas et al. [28], Divakar et al. [29], Chowdhury et al. [30], Akshai et al. [31], Kibriya et al. [32], B.V. et al. [33], Pandian et al. [34], etc., which has been conducted by utilizing deep learning and convolutional neural networks, which is a DL technique. CNN is a kind of feed-forward neural network whose power lies in the convolutional layer; there is less need to pre-process data in CNN.

This paper is divided into six different sections. Section 1 is the introduction itself. The remaining sections of the paper are arranged as follows. Section 2 describes the research methodology utilized for finding and analyzing the available existing research, research questions, and research criteria. The literature review of previously published studies is described in Section 3. Section 4 discusses the challenges of the existing approaches. Overall, observation and comparison are provided in Section 5, and this paper is concluded in Section 6.

This paper’s significance lies in its discussion of many methods that have been created to identify plant illnesses from their leaves in the domains of ML, image processing, and DL.

## 2. Methodology

This section presents information regarding the planning and selection criteria for selecting relevant papers for this review.

### 2.1. Planning

The investigation included compiling a number of journal and conference articles that were released between 2010 to 2022. First, keyword-based searches were made in scientific databases such as IEEE Xplore, SCOPUS Indexed Journal, and Google Scholar (academic search engines). In Table 1, the list of searched keywords is provided.

Using different keywords, 182 papers were extracted on which inclusion and exclusion operations were performed.

### 2.2. Conduction

**Figure 1:**
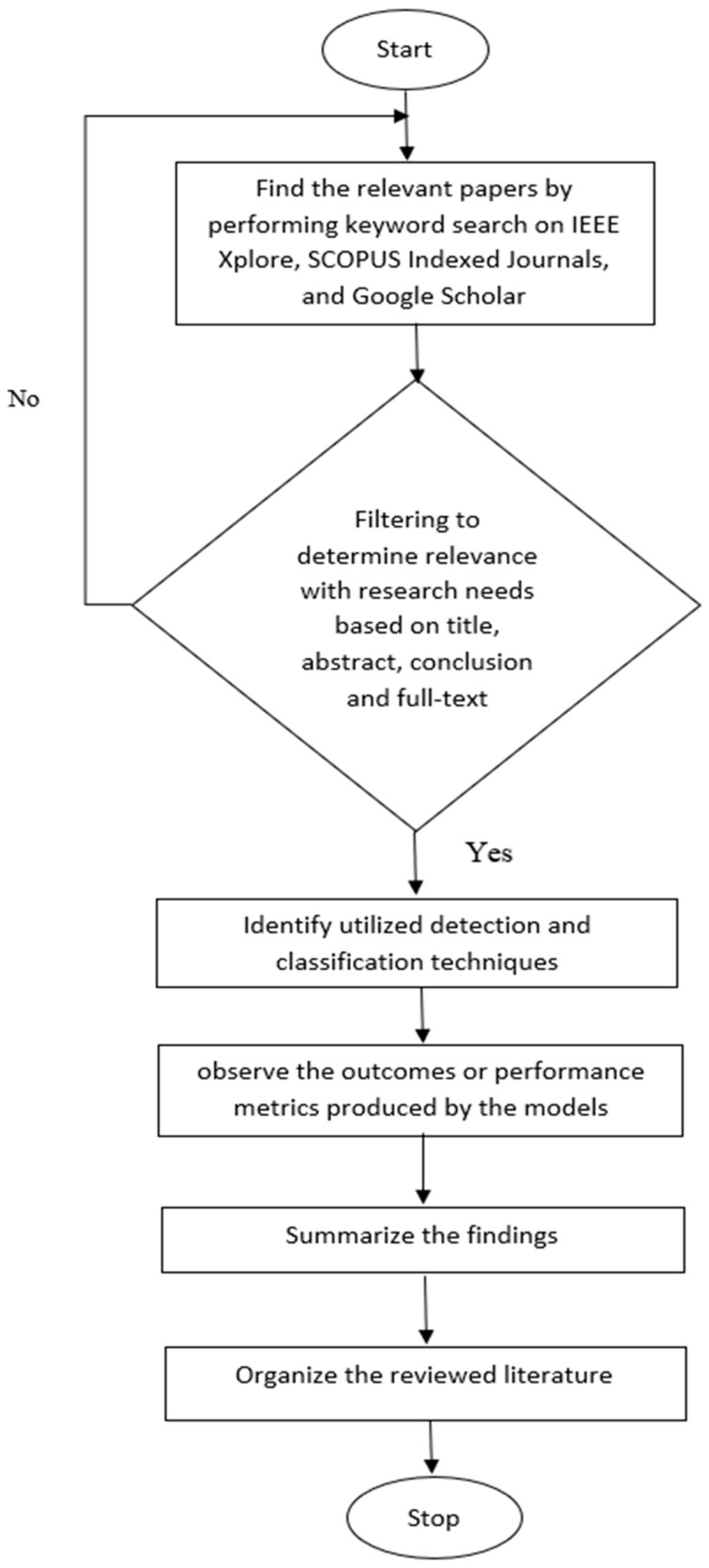
The entire method of research utilized to produce this study.

This phase focuses on reviewing and summing up the selection criteria for assessing existing models based on ML, image processing techniques, and DL, including CNN, in terms of effective disease detection in different crops and plants using different datasets. In Figure 1, the entire research method utilized to produce this study is shown.

**Figure 2:**
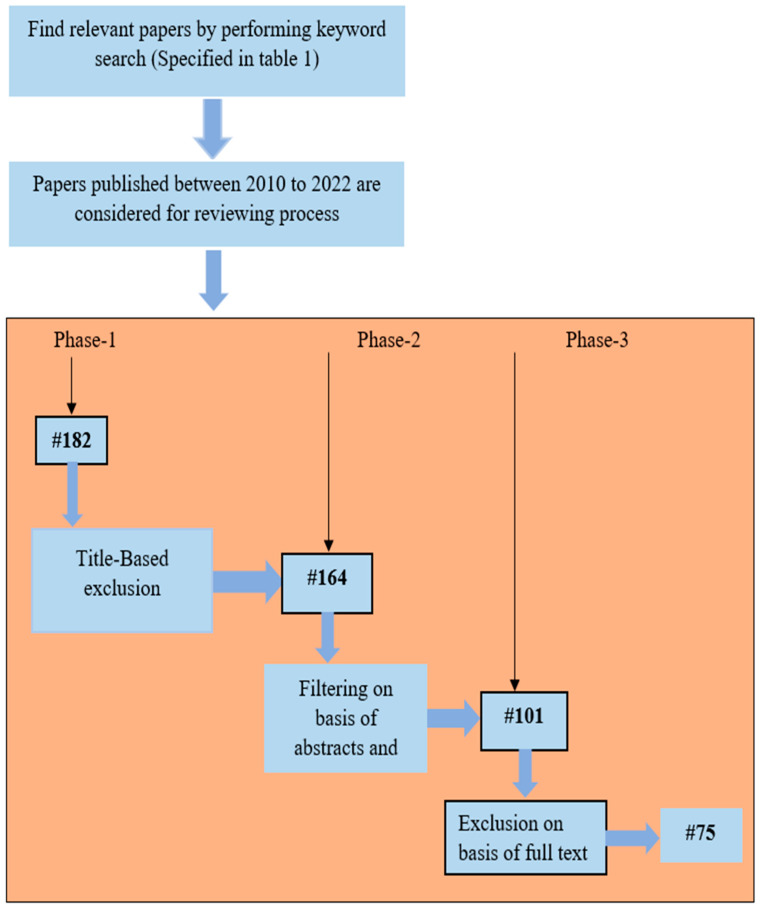
Inclusion and exclusion techniques utilized in this review.

**Figure 3:**
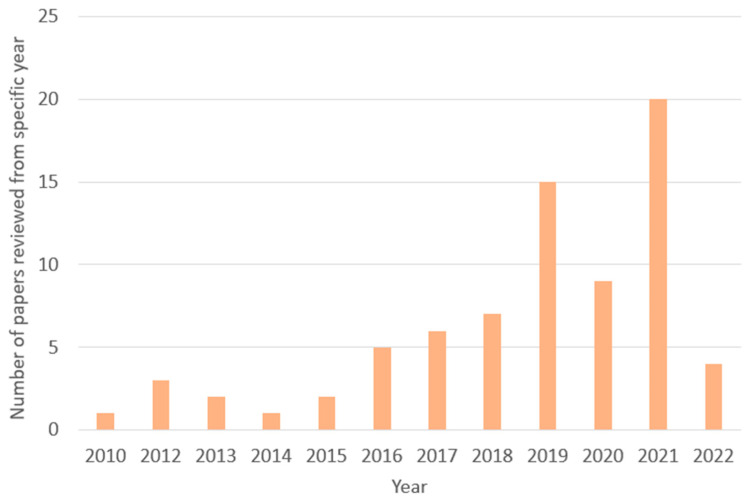
Number of papers, by year, from 2010 to 2022.

By conducting a keyword search, 182 papers on plant disease detection and classification from sources such as IEEE Xplore, SCOPUS Indexed Journal, and Google Scholar were retrieved that were published in the last 12 years from 2010 to 2022. Three stages made up the exclusion process. The retrieved data were then decreased to 164 based on their titles; publications were then eliminated based on their abstracts and conclusions; and, finally, 75 papers were found after reading the entire text. Figure 2 and Figure 3 represent the number of papers reviewed by year from 2010 to 2022.

For the purpose of writing a systematic review, ten research questions were framed, which are specified in Table 2, and a complete evaluation procedure was conducted by monitoring the existing models for the purpose of addressing research questions.

## 3. Related Work

**Figure 4:**
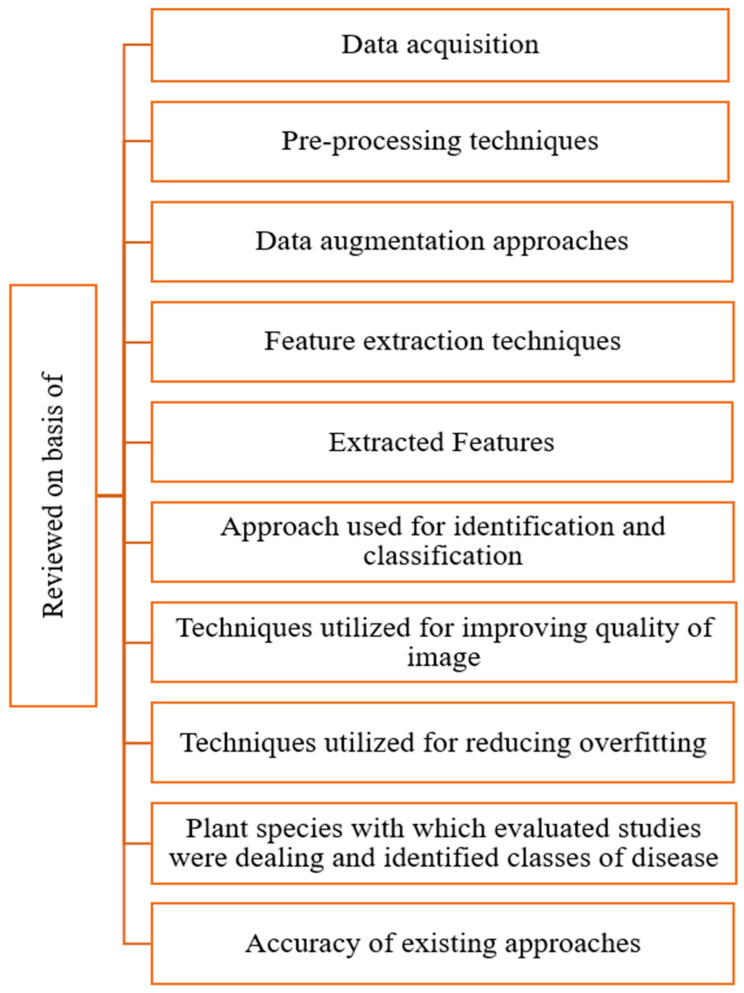
Parameters considered for the literature review.

On the basis of the data obtained from the chosen studies, the research methodology findings in this section provide answers to the research questions listed above. These automated models require significant training time, but once they are trained, they are incredibly accurate at spotting early-stage plant diseases and enabling farmers to take preventative action to lessen the effects of disease on productivity. Figure 4 shows various parameters that were considered for review. The approach that was utilized for conducting this literature review included data acquisition, pre-processing techniques, techniques for augmenting data, techniques for extracting features, different features that were extracted, techniques utilized for identification and classification, how the quality of images was enhanced, and techniques utilized for reducing overfitting of the models. The research questions (RQ1 to RQ10) listed in Table 2 are discussed in Section 3.1, Section 3.2, Section 3.3, Section 3.4, Section 3.5, Section 3.6, Section 3.7, Section 3.8, Section 3.9 and Section 3.10.

### 3.1. Discussion for RQ1: What Are the Main Sources for Collecting Data on Plants?

The primary step in the identification and categorization of leaf disease is the acquisition of plant leaf imaging data. Images of plant leaves can be taken manually using a camera device, or image data can be acquired from open-source repositories. This section involves discussion on different sources from which image data was collected by various researchers for their work. A total of 46 publications, which were identified as the sources of the data for this section, were taken into consideration for analysis.

Rumpf et al. [2] utilized images of sugar beet leaves that were cultivated in plastic pots on a commercial substrate, namely Klassmann–Deilmann GmbH (Germany), in an environment with 60 percent relative humidity, a 23 °C daytime temperature, and a 20 °C nighttime temperature. In addition to weekly fertilization with 100 mL of a Poly Crescal 0.2 percent solution, plants were watered as needed. To identify and categorize plant diseases, the study used hyperspectral reflectance-based approaches. Wang et al. [35] utilized backpropagation networks on a dataset of 185 images—acquired using a digital camera—to identify two distinct instances of wheat and grape illnesses. The collection included 50 wheat stripe rust images, 35 grape powdery mildew images, and 50 images each of grape downy mildew and wheat leaf rust. Husin et al. [36] utilized LABVIEW IMAQ Vision to collect a dataset of 107 images of chili leaves, 21 of which were healthy and 86 of which were diseased. Sannakki et al. [37] acquired images of grape leaves with a 16.1 Megapixel Nikon Coolpix P510 digital camera from various locations, including Pune, Bijapur, and Sangali, under the guidance of experts for identification. For system testing and training, the captured images were employed. The captured images were all saved in the common .jpg format. To provide a diversified environment, several images were acquired from the internet. Images were taken of leaves afflicted by powdery mildew and downy mildew, two of the most common illnesses in India. 

Es-saddy et al. [38], under the guidance of an expert, utilized a digital camera to collect image data from a number of farms. The dataset’s size was increased by downloading images from the internet and using a variety of environments. The images include leaf damage from thrips, Tuta Absoluta, and leaf miners (pest insects). Sladojevic et al. [8] acquired a dataset by searching for plants’ names and diseases. Images were collected from the internet and divided into 15 distinct categories, out of which 13 represented plant diseases to be visually identified from leaves, and the remaining 2 represented healthy leaves and background images. 

Fujita et al. [9] utilized two separate datasets that were created using cucumber leaf images provided by the Research Center of Saitama Agricultural Technology, Japan. Dataset 1 contained 7320 images, including images of leaves that were affected by seven distinct diseases and images of healthy leaves, whereas dataset 2 contained 7520 images. Dyrmann et al. [39] utilized images acquired from a real environment and six different publicly available datasets for their work. A total of 10,213 images representing 22 different species in terms of changes in illumination, resolution, and soil types were captured with hand-held mobile phones. 

Mohanty et al. [40] acquired data from a publicly available dataset (PlantVillage dataset which is available on kaggle) of 54,306 images, which were categorized into 38 distinct classes of diseased and healthy leaves that were gathered under controlled circumstances. Durmus et al. [41] utilized the PlantVillage dataset to acquire images of 10 different tomato leaf classes, out of which 9 were disease-affected leaf images and 1 was a healthy leaf image. Brahimi et al. [10] utilized an open-access repository (PlantVillage) of more than 50,000 images to gather data on around 14,800 images of tomato leaves that were afflicted by 9 distinct diseases. Fuentes et al. [11] used basic camera devices to collect data of over 5000 images from several tomato fields in Korea while taking lighting, temperature, humidity, and location into consideration. Liu et al. [42] utilized a digital camera, namely, BM-500 GE/BB-500 GE, to collect a dataset of 1053 apple leaf images from 2 apple experiment stations in China. Four distinct diseases, namely, rust, brown spot, Alternaria leaf spot, and mosaic, could be seen in the acquired images. 

Ma et al. [13] acquired image data from two open-access repositories and a digital camera. Powdery mildew, anthracnose, target leaf spots, and downy mildew were some of the symptoms represented in the dataset, which was obtained from two open-access repositories (https://plantvillage.org/and https://www.forestryimages.org/ (accessed on 14 January 2023)). Some image data were also taken using a Nikon Coolpix S3100 (digital camera) from an agricultural scientific innovation base greenhouse in Tianjin (China) under field conditions. Sardogan et al. [14] utilized a public dataset called the PlantVillage dataset to obtain 500 images of tomato leaves, including 4 distinct kinds of diseased leaves (late blight, septoria leaf spot, bacterial spot, and yellow leaf curl) and images of healthy ones, of which 100 were used for testing and 400 for training. Cap et al. [12] utilized image data provided by the Saitama Agricultural Technology Research Center in Japan, which included over 60,000 images of cucumber leaves. Behera et al. [5] acquired images of four different diseased orange samples, including those affected by melanoses, brown rot, stubborn, and citrus canker, from a dataset of the division of Agriculture and Natural Resources at the University of California. Geetharamani et al. [16] acquired image data from the PlantVillage dataset, which contains about 54,000 images divided into 38 different groups, to train and test the proposed CNN model. 

Atila et al. [43] and Too et al. [44] obtained image data from the open-source dataset, PlantVillage, which includes around 54,000 images of 14 distinct plant species. Wahab et al. [7] using a series of camera movements that had been pre-programmed, which enabled them to capture images from multiple orientations and heights. Image data of a chili plant was acquired and recorded in Matlab. KC et al. [45] acquired data from the publicly accessible PlantVillage dataset, which consists of over 82,000 images divided into 55 separate classes. Both diseased and healthy leaf were including images for the purpose of training and testing the proposed model. Haque et al. [46] collected ten thousand images using a Nikon D7200 DSLR camera, under various conditions, for four different types of guavas: fruit canker, anthracnose, fruit rot (disease-impacted), and healthy guava. Sahithya et al. [47] acquired ladies’ finger leaf images using a 1584 × 3456 resolution digital camera. 

Chen et al. [48] acquired image data of roughly 1000 images of rice and maize leaves damaged by various diseases given by the Xiamen, China-based Fujian Institute of Subtropical Botany. The image dataset was taken under various lighting circumstances and cluttered field background conditions and stored in .jpg format. Marzougui et al. [21] captured 500 images in total, 250 of which were of healthy leaves, while the other shots, which were all taken with a camera against a consistent background, were of diseased leaves. Ponnusamy et al. [49] collected image data of around 300 healthy and diseased tomato leaves from agricultural fields for a disease diagnosis model based on YOLO, considering the camera quality, impact of the number of leaves in the frame, exposure, and zoom level. Nanehkaran et al. [50] gathered about 1000 diseased leaf images of three distinct crops—rice, maize, and cucumber—in diverse manners. While images of damaged maize and rice leaves were taken from research farms in Xiamen (China), image data pertaining to cucumber leaves affected by various diseases were retrieved from the internet. All the collected images were stored in .jpg format. 

Pham et al. [51] utilized a 3096 × 3096 resolution camera to acquire a dataset of roughly 450 images of mango leaves from Giang Province, Vietnam. The dataset included three types of disease-infected leaves (powdery mildew, anthracnose, and gall midge) and one category of healthy leaves. The CNN model suggested by Selvam et al. [23] was trained and tested using image data of around 1085 lady finger leaves (healthy, disease-affected, and impacted by the overuse of fertilizers) collected from two villages in the Tiruvannamalai district. Jadhav et al. [24] gathered soybean-related image data from a number of soybean fields in Kolhapur district, Maharashtra, India. A total of 1199 photos were utilized for training the suggested CNN models, and roughly 80 images were used to test them. Sun et al. [26] collected image data from the open-source PlantVillage dataset, of which 80% were used for training and 20% for testing the proposed model. Lijo [25] acquired 10,000 diseased and healthy images of potato, mango, strawberry, grape, tomato, and pepper leaves from the plant village dataset. Both bacterial and fungal illnesses were included in this research. Chakraborty et al. [52] utilized images of 13 plant species and 17 kinds of illnesses, comprising approximately 2600 images, which were obtained from PlantDoc.

Abbas et al. [28] acquired diseased and healthy tomato leaf images from the open-source PlantVillage dataset. Wang et al. [53] acquired 3000 leaf images of various species, both healthy and disease-affected, that were gathered from the PlantVillage dataset. Divakar et al. [29] acquired image data that contained images of both diseased and healthy apple leaves, which were downloaded from a publicly accessible dataset on Kaggle. Chowdhury et al. [30] and Gonzalez-Huitron et al. [54] acquired image data of around 18,100 tomato leaves from the PlantVillage dataset. It was composed of ten classes, of which nine represented various disease-affected leaves and one contained healthy leaves. Akshai et al. [31] acquired images of about 4060 grape leaves, including both healthy and diseased leaves of various categories, from the PlantVillage dataset. Kibriya et al. [32] acquired around 10,000 tomato leaf images from the PlantVillage dataset, out of which 30% were utilized to test the suggested model, whereas 70% were used for training it. 

B.V. et al. [33] for the purpose of identifying potato and tomato leaf diseases, utilized a subset of tomato and potato leaf images from the publicly accessible PlantVillage dataset. Jain et al. [55] acquired image data for three different crops—maize, grapes, and rice—from the New Plant Diseases Dataset, which is available on Kaggle. Sujatha et al. [27] gathered images of citrus leaves using DSLR cameras under the direction of experts. Pandian et al. [34] acquired about 55,448 images of both healthy and diseased leaves of various plant species from a freely accessible dataset. Vallabhajosyula et al. [56] acquired RGB images of 14 distinct types of crop species and 38 classifications of damaged leaves from the open-source dataset PlantVillage, which was downloaded from Kaggle. Table 3 represents a summarized view of the different data acquisition sources utilized by the reviewed studies. Table 4 provides information about the sources in the real environment from which images were gathered by various researchers for their work.

#### Observation 1

**Figure 5:**
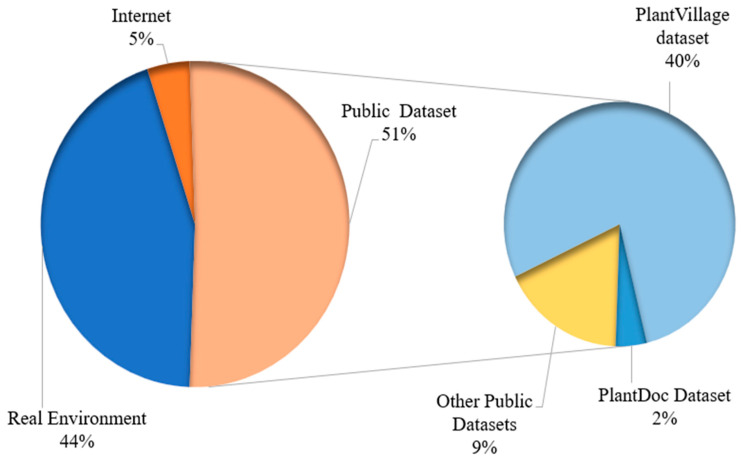
Usage of various data acquisition sources.

This observation is purely framed on the basis of discussions for RQ1 (3.1): 51% of the research under consideration acquired image data from publicly accessible datasets, while 44% employed digital cameras or other devices to collect images from the real environment, and the other 5% obtained their image data from other online sources. The primary publicly accessible datasets used in the evaluated studies were PlantVillage, PlantDoc, and other public datasets. All of the data acquisition sources are depicted in Figure 5.

### 3.2. Discussion for RQ2: What Different Pre-Processing Techniques Are Applied?

For further processing, image data were pre-processed utilizing a number of different techniques. This section involves a discussion on various pre-processing techniques that have been employed by various researchers in their work. By using “Pre-processing techniques” as a filter, 34 papers were identified for this section, of which 26 papers were chosen for analysis.

Sannakki et al. [37] pre-processed images using anisotropic diffusion to produce space-variant and non-linear changes to the original images. Khirade et al. [57] utilized various image pre-processing techniques, including image smoothing, clipping, image enhancement, color conversion, and histogram equalization, to eliminate noise from the images. Rastogi et al. [58], prior to training and testing the proposed model, pre-processed the image data that were gathered during the image acquisition phase by resizing and cropping operations. Es-saddy et al. [38] first downsized the images into a standard size using a resizing operation, and then the noise was eliminated from them using a median filter to enhance their quality. Sladojevic et al. [8] performed two pre-processing operations, including resizing, where the image was scaled into 256 × 256 pixels, and cropping, which was performed to define the regions of interest in plant leaves for improved feature extraction. 

Singh et al. [59] carried out several operations during the pre-processing stage to improve the quality of the image, including the clipping operation to extract the relevant image regions and the use of smoothing filters to improve the image’s smoothness. In order to increase the image’s contrast, image enhancement was used. In the study by Krithika et al. [60], during the pre-processing stage, pixels from grape leaf images’ edges were deleted, and RGB data collected during the data acquisitionphase were transformed into the HSV and CIELAB color spaces. Ferentinos [61] performed image size reduction and cropping as part of the pre-processing operations that were performed on collected image data to make the images 256 × 256 pixels. Ramesh et al. [4] pre-processed the collected images to make them all the same size. Behera et al. [5] utilized two techniques for pre-processing images. The first technique used was image enhancement, which increased the contrast in the images and drew attention to any hidden details that may have been there, while another technique used was CIELAB color space, which shortened the computing time. Francis et al. [62] downsized images to 64 × 64 pixels using the resizing and cropping pre-processing techniques. 

Devaraj et al. [63] utilized different MATLAB algorithms throughout the pre-processing step to downsize images, improve the contrast, and transform the RGB images into greyscale. Wahab et al. [7] utilized MATLAB’s reb2gray function in the pre-processing step to convert RGB format images into grayscale while retaining luminance and removing hue and saturation. Howlader et al. [64] used Python code in pre-processing for the purpose of scaling all of the acquired images to 256 × 256 pixels. Sharma et al. [18] performed different pre-processing operations on images to enhance the quality of an image by eliminating noise from it. This was performed by enhancing compactness, changing brightness, extracting noise, and converting to another color space. Sahithya et al. [47] performed a resizing operation on the image to convert all of the images of the same standard size. Jadhav et al. [24] converted images into two different dimensions for AlexNet and GoogleNet. For AlexNet, a total of 649 images of soybean were pre-processed into dimensions of 227 × 227 × 3, whereas 550 images of soybean leaf samples underwent the same pre-processing for the proposed GoogleNet framework, resulting in dimensions of 224 × 224 × 3. Chen et al. [48], for the purpose of creating images of the same size, blackened shorter sides of images during the pre-processing step. Pham et al. [51], during the pre-processing stage, downscaled the images to a lower resolution, and pixel intensities were adjusted using contrast enhancement. Lijo [25] scaled images to 256 × 256 pixels during the pre-processing stage.

Chowdhury et al. [30] utilized various pre-processing operations. Operations such as resizing and normalization were carried out in the pre-processing step. All collected images were downsized to 224 × 224 for various EfficientNet approaches, while they were all converted to 256 × 256 for U-net segmentation techniques. In addition to resizing, the means and standard deviations of the images in the dataset were computed in order to normalize the z-score data. Kibriya et al. [32] utilized two distinct image processing methods, namely, resizing and denoising. The images were denoised using the Gaussian Blur filter, and all of the collected data were scaled to 225 × 225. Chouhan et al. [65] pre-processed image data using the resizing, restoration, and image enhancement techniques. Malathy et al. [1] pre-processed image data are utilizing image resizing and image restoration, which lessen image noise and improve the image’s sharpness. 

Jain et al. [55] improved the images’ quality by employing a 3 × 3 Gaussian filter to remove noise from the image during pre-processing. Ashwinkumar et al. [66] utilized bilateral filtering, a non-linear filtering technique, during the pre-processing stage to enhance the quality of an image by eradicating noise from the acquired image data. Table 5 shows a summarized view of the various pre-processing methods applied in different reviewed studies.

#### Observation 2

**Figure 6:**
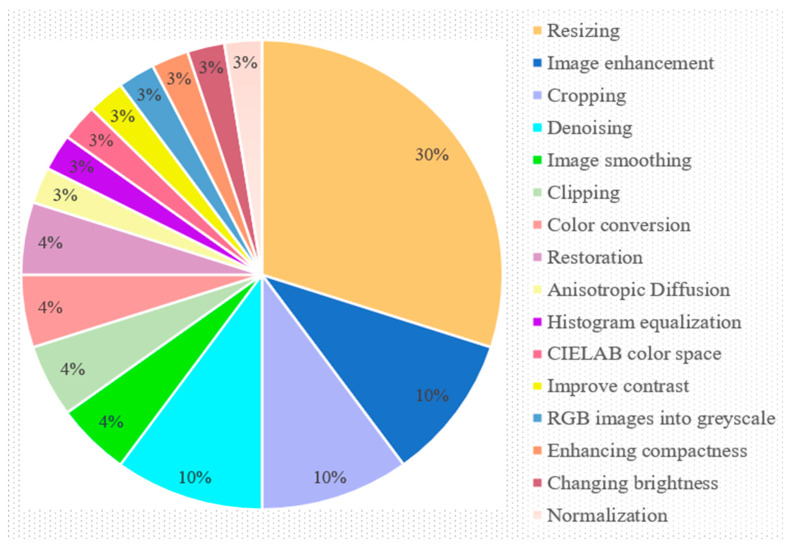
Usage graph of different pre-processing techniques (% in descending order).

This observation is solely based on RQ2 (3.2) discussions. The studies under evaluation included a variety of pre-processing methods, including scaling, clipping, smoothing, anisotropic diffusion, cropping, denoising, CIELAB color space, contrast improvement, converting RGB images to greyscale, increasing compactness, restoration, and normalizing. Figure 6 shows how various pre-processing methods are generally utilized. First, 30% of the papers that were examined used resizing to pre-process images, while the image improvement, cropping, and denoising operations were utilized by 30% of examined studies combinedly, 10% of each respectively. In the publications that were reviewed, 4% of the restoration, color conversion, clipping, and smoothing procedures were used individually, while 3% of the other pre-processing approaches were utilized individually.

### 3.3. Discussion for RQ3: What Different Techniques Are Used for Data Augmentation?

Various data augmentation approaches can be utilized to enhance the dataset’s image count in order to improve accuracy. In this section, techniques utilized by various researchers in their works to increase the size of dataset have been discussed. Twenty-eight papers were selected which used data augmentation for this section, and are currently being considered for analysis.

Sladojevic et al. [8] utilized three different operations, namely, rotations, 3 × 3 transformation matrix-based perspective transformation, and affine transformations for augmenting images. Fujita et al. [9] utilized three different augmentation techniques—image shifting, mirroring, and image rotation—to expand the dataset. Dyrmann et al. [39] utilized rotation and mirroring techniques to expand the training dataset to 50,864 images (eight times the number of original images). Fuentes et al. [11] increased the image count in the training dataset through the use of the two image augmentation approaches, namely, geometrical transformation and intensity transformations. While procedures including image scaling, cropping, rotation, and horizontal flipping were carried out during geometrical transformation, intensity transformation dealt with noise, color, brightness enhancement, and contrast. Ma et al. [13] utilized the rotation and flipping operations to increase the amount of image data. Images in the dataset were rotated by 90, 180, and 270 degrees during the rotation process, but during the flipping operation, images were flipped in both the horizontal and vertical directions. Cap et al. [12] increased the dataset’s image count utilizing cropping (from the center) and rotation (clockwise) operations. 

Kobayashi et al. [67] utilized several augmentation techniques, including rotation, shear conversion, cutout, and horizontal and vertical direction, to expand the size of the dataset in order to improve detection accuracy. Geetharamani et al. [16] utilized augmentation operations such as flipping, principal component analysis, rotation, scaling, noise injection, and gamma correction to expand the dataset’s size to approximately 61,400 images. Zhang et al. [17] utilized intensity transformations and geometric transformations to increase the number of images. There were five approaches used for the intensity transformations: contrast enhancement, color jittering, PCA jittering, blur (radial), and brightness enhancement. Images were enlarged, cropped, rotated, and flipped in geometric transformations (horizontally and vertically). Adedoja et al. [15] utilized different combinations of data augmentation techniques, including RandomRotate, RandomFlip, and RandomLighting, which added to images so that they could be evaluated from various perspectives. KC et al. [45] augmented the image data using cropping, flipping, shifting, rotating, and combining these techniques. 

Haque et al. [46] applied several augmentation methods, including flipping (horizontal flip), zooming, shifting (height and breadth), rotating, nearest fill, and shearing, to lessen the overfitting of the guava images in the dataset. Coulibaly et al. [19] utilized four different operations by which images were augmented, namely, rescale, flipping, shift, and zoom. Ji et al. [20] increased the number of images of grape leaves with the aid of various data augmentation techniques, including rotation, zooming, flipping, shearing, and color changing. Chen et al. [48] utilized rotation, flip, scaling, and translation operations to increase the amount of image data in the utilized dataset. Kannan E et al. [68] utilized two different operations to increase the size of the dataset. Using RandomResizedCrop, where images were cropped to a size between 0.08 and 1; RandomRotation by 30 degrees; and both of these techniques together, the dataset was increased by fourfold. 

Marzougui et al. [21] utilized the “Keras Image Data Generator” class, and operations such as flip, rotation, and shift were carried out to increase the amount of image data. Images were rotated by 30 degrees and flipped horizontally, the fill mode was set to nearest, and shift operations were carried out both vertically and horizontally for better results. Selvam et al. [23] performed five different augmentation operations, namely, rotation, flipping (horizontally), shear, zoom, and shift (height, width), to increase the count of images of lady’s finger leaves. Lijo [25] utilized rotation, contrast enhancement, brightness enhancement, and noise reduction to increase the amount of image data. Divakar et al. [29] utilized the synthetic minority oversampling technique (SMOTE) to increase the count of images in the dataset in a balanced manner. Chowdhury et al. [30] performed three affine transformation operations—scaling, rotation (clockwise and anticlockwise), and translation (5% to 20% vertically and horizontally)—for the purpose of increasing image data. 

Akshai et al. [31], to enhance the size of the dataset while reducing overfitting, utilized different augmentation techniques, such as rotation, shifting, and zooming. Gonzalez-Huitron et al. [54] performed horizontal flipping and four-angle rotation throughout the augmentation process. B.V. et al. [33] utilized the flip operation for the purpose of increasing the count of images in the dataset. Chelleapandi et al. [69] carried out five different data augmentation operations, including rotation, filling, flipping, zooming, and shearing, using the Keras library to enhance the dataset. Pandian et al. [34] utilized neural style transfer, position and color augmentation, deep convolutional generative adversarial network, and principal component analysis to increase the number of images from 55,448 to 234,008. Vallabhajosyula et al. [56] performed four different augmentation approaches— scaling, translation, rotation, and image enhancement—to increase the size of the dataset and to reduce overfitting. Table 6 shows a summarized view of the various data augmentation techniques utilized in different evaluated studies.

#### Observation 3

**Figure 7:**
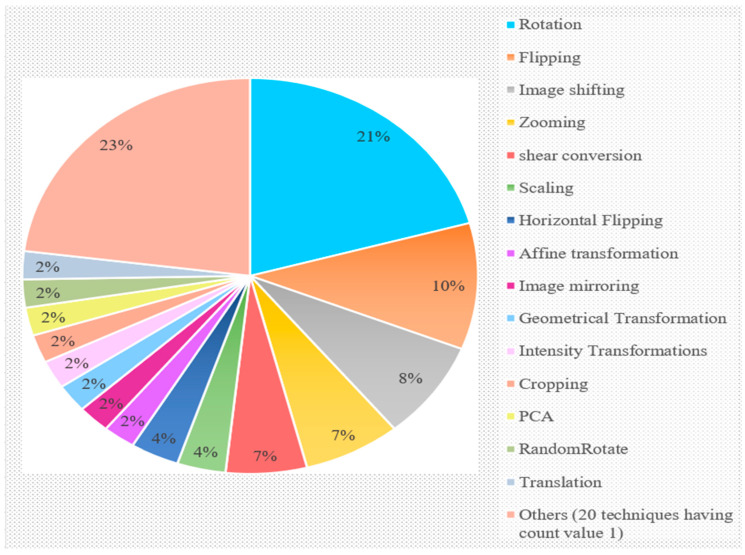
The deployment percentage for various augmentation methods.

This observation is purely based on discussions of RQ3 (3.3). Numerous data augmentation methods, such as rotation, mirroring, cropping, flipping, PCA (color augmentation), zooming, shifting, scaling, RandomRotate, translation, etc., were used in the reviewed studies for increasing the dataset’s image count. The overall utilization of the various augmentation techniques used in the reviewed studies is shown in Figure 7.

From the figure, it is clearly evident that rotation was much more frequently utilized for increasing the dataset’s image count than other methods (21%), while flipping came in second, with a value of 10%. Mirroring was employed for augmentation in 8% of the investigations, while zooming and shearing were utilized in 7% of the research, respectively. Additionally, 2% of the evaluated studies utilized affine transformation, mirroring, geometrical transformation, intensity transformation, cropping, PCA, RandomRotate, and translation, whereas only 1% of the other 20 techniques were used individually.

### 3.4. Discussion for RQ4: What Kinds of Feature Extraction Methods Are Employed?

This section involves a discussion of various feature extraction methods that were utilized by various researchers in their works. Twenty-six papers were found by using feature extraction techniques for filtering, of which nineteen papers are utilized for analysis in this section.

Husin et al. [36] take color space into account; using this, the illumination from images can be reduced, allowing for an effective determination of whether a leaf is from a chili plant or not. Images were extracted for information pertaining to color matching, color identification, and color information. Dubey et al. [3] utilized the color coherence vector, global color histogram, complete local binary pattern, and local binary pattern methods for retrieving/extracting features. Sannakki et al. [37] utilized the color co-occurrence method for extracting texture features. Rastogi et al. [58] utilized the gray level co-occurrence matrix (GLCM) for extracting features. Es-saddy et al. [38] extracted three distinct categories of features, namely, shape, color, and texture. While textural features were retrieved using GLCM, color features were extracted using the color histogram, color structure descriptor, and color moments (skewness, mean, and standard deviation). The complexity, area, circularity, and perimeter were used as shape features. Singh et al. [59] extracted features using the color co-occurrence approach. Krithika et al. [60] extracted texture features by utilizing GLCM. Ramesh et al. [4] utilized the histogram of oriented gradients (HOG) as a feature extraction method for creating feature vectors. 

Behera et al. [5] utilized GLCM for extracting textural features. Tulshan et al. [6] and Devaraj et al. [63], using GLCM, retrieved the relevant features needed for classification. Kumari et al. [70] extracted features from the segmented cluster that contained the leaf segment afflicted by the disease after converting the images to greyscale. Color and texture (extracted using GLCM), two distinct types of features, were retrieved from images in the works by Wahab et al. [7] and Sahithya et al. [47]. Chen et al. [71] utilized RESNET18 (CNN) and a task-adaptive procedure for extracting features. Chouhan et al. [65] utilized scale-invariant feature transform for extracting features. Jain et al. [55] extracted two main kinds of information from the images: texture features and color features. To extract color features, the skewness, standard deviation, kurtosis, and mean of the color moment equation were used. With the use of a GLCM, the second class of features was extracted. Pandian et al. [34] utilized several optimal convolutional layers for extracting features. Ashwinkumar et al. [66] utilized the MobileNet model, which is based on CNNs, for extracting the necessary information from the images. Table 7 shows a summarized view of the various feature extraction techniques applied in different reviewed studies.

#### Observation 4

This discussion is purely based on RQ4 (3.4). The evaluated studies utilized various approaches for extracting features, namely, GLCM, HOG, color co-occurrence, global color histogram, etc. 

**Figure 8:**
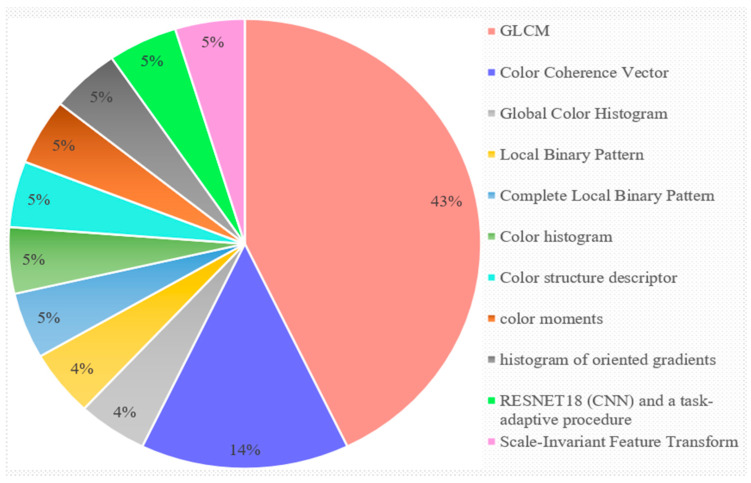
Utilization of different feature extraction techniques in % (% in descending order).

Figure 8 makes it clear that 43% of the examined research used GLCM to extract features, with color coherence vector coming in second place, with 14% of the total. Additionally, only 4% of the examined research used the global color histogram and local binary pattern, whereas 5% of the studies used the complete local binary pattern, color histogram, color structure descriptor, color moments, HOG, RESNET18, and task-adaptive process. The overall utilization of the different feature extraction techniques in the reviewed studies is shown in Figure 8.

### 3.5. Discussion for RQ5: What Are the Typical Attributes That Are Used or Extracted?

This section contains information about different features that were extracted during the feature extraction process. Using extracted features to filter the pool of publications, 20 papers were found for this section, out of which 14 were used for analysis and are represented in Table 8.

#### Observation 5

**Figure 9:**
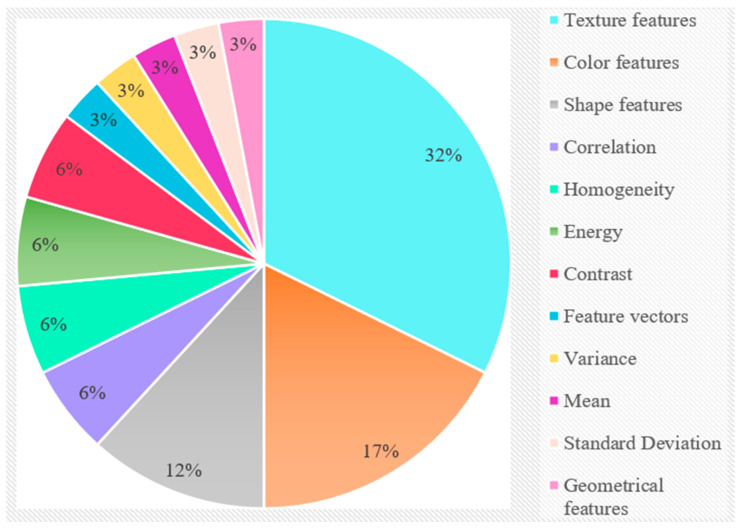
Utilization of various extracted features in % (% in descending order).

This observation is purely based on the discussion for RQ5 (3.5). The reviewed studies extracted various features during the feature extraction stage, namely, color features, shape features, correlation, texture features, energy, variance, mean, geometrical features, and standard deviation. Figure 9 shows the utilization, in percentages, of the different extracted features used in the evaluated studies.

The chart shows that 32% of the evaluated studies extracted texture features, 17% color features, and 12% shape features during the feature extraction stage. This indicates that the majority of the examined studies extracted texture features. In addition, 6% of the research that was reviewed extracted each feature, namely correlation, homogeneity, energy, and contrast, while 3% of the studies retrieved feature vectors, variance, mean, geometrical features, and standard deviation.

### 3.6. Discussion for RQ6: What Automated Systems Have Been Implemented for Identifying and Categorizing Plant Diseases?

This section involves discussion on different machine learning- and deep learning-based approaches that were utilized by various researchers in their works for the identification and classification of diseases. By sifting through them using existing automated algorithms created for identifying and categorizing plant diseases, 45 publications were found to be relevant to this subject. For analysis, 37 papers were considered.

Rumpf et al. [2] utilized an SVM on hyperspectral data for the purpose of identifying illnesses from sugar beet plant leaves, such as powdery mildew, sugar beet rust, and cercospora leaf spot. Wang et al. [35] employed backpropagation networks (BPNN) to recognize two distinct diseases in grape leaves and two different types of diseases in wheat. To identify disease in chili leaves, image processing techniques were utilized by Husin et al. [36]. Dubey et al. [3] employed multi-class SVM for recognizing and categorizing three diseases, namely, apple rot, apple blotch, and apple scab, which affect apples. Mahlein et al. [72] examined the leaves of sugar beet plants to identify three different plant illnesses using spectral disease indices. Sannakki et al. [37] utilized a feed-forward back propagation neural network (BPNN) for identifying powdery mildew and downy mildew from grape leaves. Es-saddy et al. [38] employed a serial combination of two support vector machines for identifying different types of damage to leaves by Tuta absoluta, leaf miners, and thrips (pest insects), along with late blight and powdery mildew (pathogen symptoms). Fujita et al. [9] used a CNN to identify seven distinct illnesses from cucumber leaves using CNN. Durmus et al. [41] employed two types of deep learning models, namely, SqueezeNet and AlexNet, for detecting illnesses, including leaf mold, bacterial spot, early blight, septoria leaf spot, mosaic virus, target spot, late blight, yellow leaf curl, and spider mites, from tomato leaves. Brahimi et al. [10] utilized CNN to identify nine distinct diseases from tomato leaves. 

Liu et al. [42] employed AlexNet’s deep CNN to recognize mosaic, rust, alternaria leaf spot, and brown spot in apples. Ferentinos [61] employed DL-based CNN models for identifying diseases in 25 distinct plant species. Ramesh et al. [4] utilized the random forest as a classifier in order to detect diseases in papaya leaves. Ma et al. [13] utilized deep CNN to identify downy mildew, anthracnose, powdery mildew, and target leaf spots from cucumber leaves. Sardogan et al. [14] employed a CNN model based on learning vector quantization (LVQ) to detect and categorize four distinct illnesses in tomato leaves. Behera et al. [5] utilized SVM with K-means clustering to identify four different diseases in oranges (brown rot, citrus canker, stubborn, and melanoses), while fuzzy logic was used to determine the severity of each disease. Geetharamani et al. [16] deployed a nine-layer deep CNN to detect illnesses in 13 different plant species. Francis et al. [62] employed a CNN for the purpose of identifying illness from leaves of the tomato and apple species. Kumari et al. [70] applied image processing techniques and neural networks for the purpose of identifying illnesses in cotton and tomato leaves. 

Zhang et al. [17] utilized a CNN with GAP (global average pooling) for detecting several diseases from cucumber leaves, such as gray mold, anthracnose, powdery mildew, downy mildew, black spot, and angular leaf spot. Wahab et al. [7] employed SVM (support vector machine) to locate the cucumber mosaic virus in the chili leaf plant. Adedoja et al. [15] employed NASNet for the identification of diseases. Howlader et al. [64] utilized deep CNN to detect several illnesses, including algal leaf spots, rust, and whitefly, from guava leaves, while Haque et al. [46] employed a convolutional neural network for detecting fruit rot, anthracnose, and fruit canker from the same species. Sahithya et al. [47] utilized a support vector machine (SVM) and an artificial neural network (ANN) for detecting three different illnesses from lady finger leaves, including powdery mildew, leaf spots, and yellow mosaic vein. 

Coulibaly et al. [19] utilized transfer learning with feature extraction to identify mildew in pearl millet. Ji et al. [20] employed UnitedModel (CNN) for the purpose of diagnosing three diseases from grape leaves, namely isariopsis, black rot, and esca. Jadhav et al. [24] employed two types of CNNs, AlexNet and GoogleNet, for the purpose of detecting three different illnesses, namely, frogeye leaf spot, brown spot, and bacterial blight, from soybean leaves. Sun et al. [26] applied the DM (discount momentum) deep learning optimizer for the purpose of identifying diseases of 26 distinct classes from 14 different crops. Shrestha et al. [22] utilized a CNN for the detection of different diseases from three different species—potato, tomato, and bell pepper—including early blight and late blight from potato, bell paper bacterial spot, tomato target spot, tomato mosaic virus, tomato yellow leaf curl virus, tomato bacterial spot, late blight and early blight from tomato, tomato leaf mold, tomato spider mites, and tomato septoria leaf spot. Bedi et al. [73] employed a hybrid technique based on CNN and convolutional autoencoders for the purpose of identifying bacterial spot disease from peach leaves. 

Abbas et al. [28] applied DenseNet to synthetic images produced by the Conditional Generative Adversarial Network in order to detect various diseases from tomato leaves. Chen et al. [71] utilized LFM-CNAPS (local feature matching conditional neural adaptive processes), which was developed on the basis of meta-learning, for the detection of 60 distinct diseases from 26 different plants. Akshai et al. [31] employed three distinct models based on convolutional neural networks, namely, VGG, DenseNet, and ResNet, for detecting the black rot, leaf blight, and esca diseases from grape leaves. Kibriya et al. [32] utilized GoogleNet and VGG16 for the purpose of identifying three different diseases in tomato leaves. Sujatha et al. [27] utilized three ML and three DL approaches to classify diseases. Ashwinkumar et al. [66] employed optimal mobile network-based CNN for identifying leaf mold, early blight, target spot, and late blight from the leaves of tomatoes. Table 9 shows a summarized view of the different classification techniques utilized by various researchers for classifying plant diseases.

#### Observation 6

**Figure 10:**
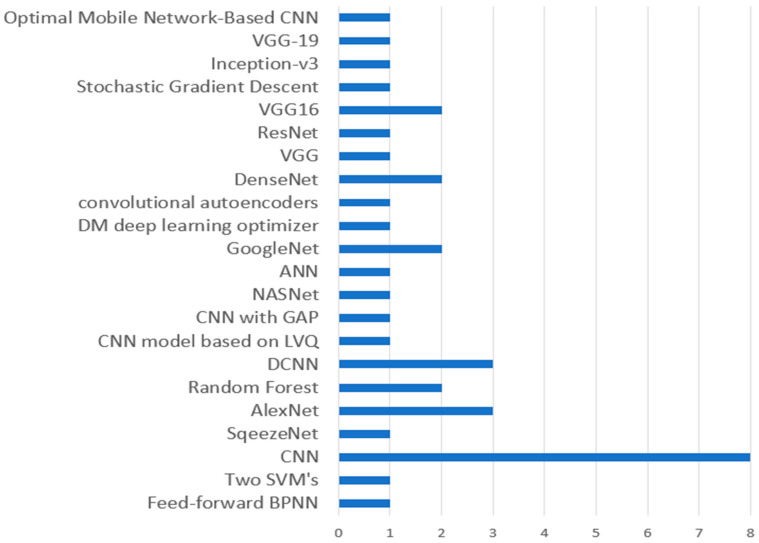
Various techniques utilized for classification.

This observation is solely based on the RQ6 discussion (3.6). As shown in Figure 10, the evaluated studies utilized numerous approaches for classifying plant diseases, including SVM, BPNN, multi-class SVM, SqueezeNet, AlexNet, ANN, VGG-19, ResNet, DenseNet, and others. The figure illustrates that while SVM was employed in five studies for identifying plant diseases, eight of the evaluated publications utilized convolutional neural networks. Secondly, three studies that were examined used DCNN and AlexNet. Third, the VGG16 model, DenseNet, and GoogleNet were utilized in two reviewed studies. Additionally, all other diagnosis methods, such as backpropagation, multi-class SVM, feed-forward BPNN, two SVMs, SqueezeNet, CNN with GAP, CNN based on LVQ, NasNet, DM optimizer, autoencoders, VGG, ResNet, inception-v3, and optimal mobile network-based CNN, were each used once by the evaluated studies.

### 3.7. Discussion for RQ7: What Analytical Techniques Are Employed for Improving Image Quality?

This section involves discussion on the techniques that were utilized by various researchers for improving the quality of the images. By filtering them using methods for improving image quality, 16 publications were found for this section. Eleven papers were finally considered for the study. 

Wang et al. [35] denoised images of wheat and grape leaves with diseased symptoms using a median filter to improve the image quality. Thangadurai et at. [74] utilized two techniques for image enhancement, namely, color conversion and histogram equalization, to improve the quality of the images. Images from RGB sources were changed to greyscale using color conversion. The images became clearer after histogram equalization. Khirade et al. [57] enhanced the image quality by histogram equalization. Es-saddy et al. [38] and Singh et al. [59] increased the quality of the images during the pre-processing stage. Krithika et al. [60] utilized the following formula during the pre-processing step to improve the greyscale images:

S_k,l_ = (T_k,l_ − min(T))/(max(T) − min(T)), where T is the original pixel value, S is the new pixel value, and (k,l) are the indices of the pixels.

Tulshan et al. [6] enhanced the quality of the images that were taken from the dataset, which were utilized as inputs during the pre-processing stage. Malathy et al. [1] performed pre-processing following image data collection to increase the image quality. Cap et al. [75] utilized LeafGAN, which significantly improved the quality of the images generated during the data augmentation stage, which also improved the proposed model’s overall performance. Vallabhajosyula et al. [56] focused on the images’ brightness and contrast during the pre-processing stage, which boosted their quality. Ashwinkumar et al. [66] employed a bilateral filter to enhance the quality of the images by removing noise from them. In the pre-processing stage, plant leaf images were used as the input images for a bilateral filter, which improved the image quality by eliminating noise. Table 10 represents a summarized view of the various techniques applied in different studies for improving the quality of the images.

#### Observation 7

**Figure 11:**
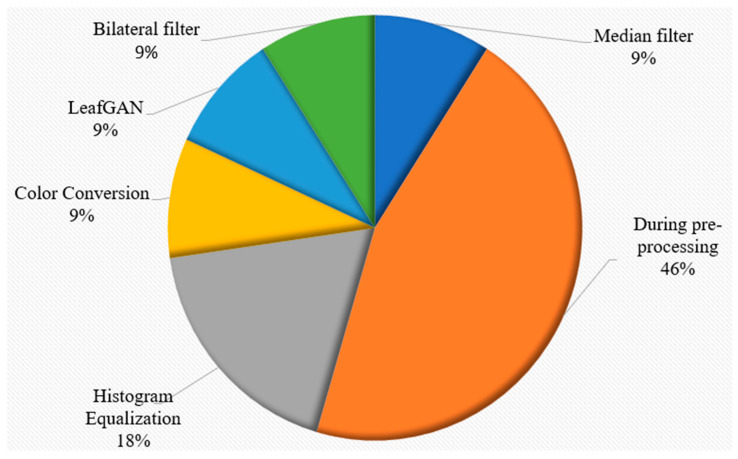
Percentages of techniques used for enhancing image quality.

This observation is purely based on the basis of discussion for RQ7 (3.7). In the evaluated studies, the quality of the images was improved using various filters, namely, bilateral and median, by histogram equalization during pre-processing and color conversion. Figure 11 was created on the basis of the information in Table 9.

### 3.8. Discussion for RQ8: What Are the Techniques Utilized for Reducing/Removing Overfitting?

When there is a significant discrepancy in the accuracy values which a model produces for training and testing datasets, it is said to overfit. In this section, the different techniques which were employed by different authors to reduce overfitting are discussed. Twenty-four publications were found for this section after filtering them using techniques to reduce overfitting. Finally, 21 papers were taken into account for analysis.

Sladojevic et al. [8] introduced some distortion to the images during augmentation to prevent overfitting. Fujita et al. [9] utilized rotation and flipping operations for data augmentation to lessen overfitting. Durmus et al. [41] applied activation function layers to increase the model’s non-linearity, while dropout layers and pooling layers were used to lessen overfitting. Fuentes et al. [11] performed extensive data augmentation in order to prevent overfitting. Liu et al. [42], by using image processing approaches (expanding the training dataset’s image count), response-normalizing layers (which enabled local normalization), and swapping out some fully connected layers for convolution layers, lessened the overfitting of the model. Ma et al. [13] minimized overfitting by expanding the dataset of cucumber leaf images using data augmentation techniques. Geetharamani et al. [16] introduced distorted images to the training dataset during image transformation to avoid overfitting. Francis et al. [62] avoided overfitting by setting the dropout value at 0.25. Ji et al. [20] employed several approaches, including an early stop mechanism, data augmentation techniques, and dropout, to minimize the overfitting of the model. Howlader et al. [64] mitigated overfitting by using the ReLU activation function and data augmentation approaches. The formula for the ReLU activation function was given as:

F(N) = Max(0, N), where N refers to the number of neurons. 

Coulibaly et al. [19] utilized the concept of an early stopping strategy to reduce overfitting. Lijo [25], Abbas et al. [28], Pandian et al. [34], Chen et al. [48], Vallabhajosyula et al. [56], and Kannan E et al. [68] reduced the overfitting of the model using data augmentation techniques. Bedi et al. [73] employed the concept of early halting, and the patience value was set to 5 to prevent model overfitting. Wang et al. [53] utilized 1 × 1 convolution to decrease overfitting. Chen et al. [71] utilized forward propagation in order to avoid overfitting. Chowdhury et al. [30] utilized GAP for the purpose of reducing overfitting. Table 11 shows a summarized view of the various techniques applied in different research for reducing or removing overfitting.

#### Observation 8

**Figure 12:**
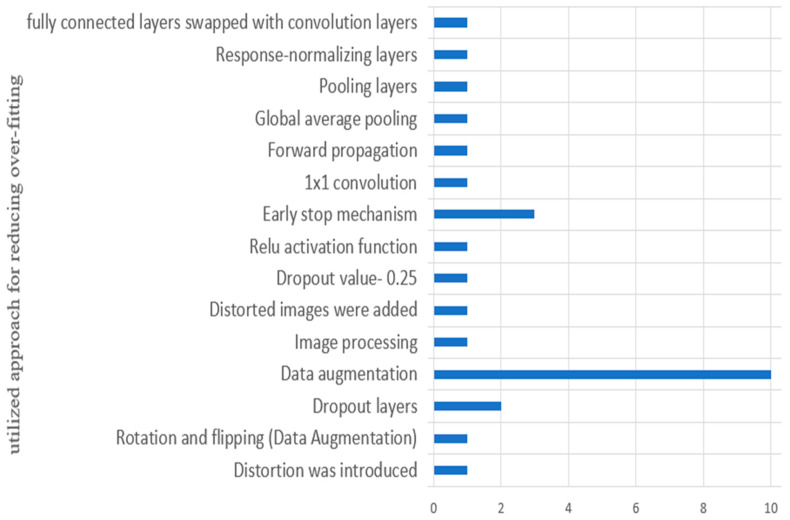
Techniques utilized for reducing overfitting.

This observation is purely based on the basis of discussion for RQ8 (3.8). In the evaluated studies, overfitting was reduced by various approaches, such as by adding distortion to images, data augmentation, global average pooling (GAP), response-normalizing layers, pooling layers, the early stop mechanism, etc. Figure 12 shows various approaches utilized for reducing the overfitting of the model. Figure 12 shows that in various evaluated studies, overfitting was reduced by data augmentation.

### 3.9. Discussion for RQ9: What Are the Different Plant Species That the Evaluated Research Is Based on, and What Classes of Diseases Have Been Found by the Evaluated Studies?

This discussion focuses on the disease classes that were discovered in the specific plant species on which the utilized reviewed studies were based.

The model used by Rumpf et al. [2] and Mahlein et al. [72] diagnosed three diseases, namely, sugar beet rust, Cercospora leaf spot, and powdery mildew, from the leaves of sugar beet plants. Wang et al. [35] suggested an approach which detected diseases from two species, namely, grapes and wheat. Downy and powdery mildew were detected in grapes, whereas leaf rust and stripe rust were found in wheat. Dubey et al. [3] suggested an approach which diagnosed apple rot, apple scab, and apple blotch from images of apples [3]. The proposed model of Sannakki et al. [37] detected two distinct classes of mildew, namely, powdery and downy, from images of grape leaves. Es-saady et al. [38] diagnosed diseases caused by pest insects (thrips, leaf miners, tutaabsoluta) and pathogens (early and late blight, powdery mildew) from leaf images. Fujita et al. [9] proposed a model which identified a total of seven distinct classes of diseases from cucumber leaf images, of which four classes were caused by mosaic viruses, including zucchini yellow, cucumber mosaic virus, watermelon mosaic virus, and kyuri green mottle mosaic virus. Three classes were caused by other viruses, including melon yellow spot virus, cucurbit chlorotic yellows virus, and papaya ring spot virus. 

Durmus et al. [41] and Brahimi et al. [10] proposed a model which was utilized for identifying nine classes of diseases, namely, leaf mold, early and late blight, yellow leaf curl virus, bacterial spot, septoria leaf spot, mosaic virus, target spot, and spider mites, from images of tomato leaves. Liu et al. [42], using the AlexNet-based model, diagnosed four distinct classes of diseases, namely, rust, alternaria leaf spot, mosaic, and brown spot, from the leaves of apples. Ferentinos [61] suggested an approach for the purpose of recognizing 58 kinds of diseases from leaf images of 25 different plant species. Ramesh et al. [4] presented a method for identifying healthy and unhealthy papaya leaves. Ma et al. [13] proposed a model which diagnosed four distinct categories of cucumber diseases, namely, target leaf spots, downy and powdery mildew, and anthracnose, from cucumber leaf images. Sardogan et al. [14] presented a model for identifying four groups of diseases from images of tomato leaves, including septoria spot, bacterial spot, tellow curved, and late blight. Behera et al. [5] proposed an approach for detecting brown rot, citrus canker, melanoses, and stubbornness from images of oranges. Geetharamani et al. [16] suggested a technique for identifying 38 classes from images of the leaves of 13 distinct plant species. Francis et al. [62] suggested an approach for categorizing healthy and diseased leaves of two species, namely, apple and tomato. 

Ji et al. [20] presented a UnitedModel for diagnosing three classes of grape diseases, namely, isariopsis leaf spot, esca, and black rot, from images of grape leaves. Kumari et al. [70] proposed a model for diagnosing diseases from images of cotton and tomato leaves. It identified two classes of cotton diseases, target spot and bacterial leaf spot, and two species of tomato diseases, namely, leaf mold and septoria leaf spot. Zhang et al. [17] suggested an approach which detected six distinct classes of cucumber diseases. The proposed model of Wahab et al. [7] identified cucumber mosaic virus from images of leaves of the chili plant. Haque et al. [46] presented a methodology for diagnosing fruit rot, anthracnose, and fruit canker from images of guava, whereas Howlader et al. [64] proposed an approach for identifying rust, algal leaf spot, and whitefly from images of guava leaves. Sahithya et al. [47] diagnosed three distinct classes of diseases, namely, powdery mildew, leaf spot, and yellow mosaic vein, from images of lady finger leaves. Coulibaly et al. [19] presented an approach for the purpose of identifying mildew in pearl millet. Jadhav et al. [24] proposed a methodology which identified brown spots, bacterial blight, and frogeye leaf spots from images of soybean leaves. 

Kannan E et al. [68] diagnosed diseases, namely, yellow leaf curl, septoria leaf spot, early blight, mosaic virus, and bacterial spot, from tomato leaf images. Sun et al. [26] proposed a model which identified 26 classes of disease from the leaves of 14 plant species. Pham et al. [51] identified three types of diseases from images of mango leaves, including powdery mildew, gall midge, and anthracnose. Shrestha et al. [22] proposed a model that diagnosed twelve classes of disease from the leaves of three species, namely, potato (two classes), tomato (nine classes), and bell pepper (one class). The 12 classes of diseases included late and early blight (potato); bell pepper bacterial spot; and, in tomato plants, yellow leaf curl virus, target spot, mosaic virus, septoria leaf spot, early blight, bacterial spot, leaf mold, late blight, and spider mites. Bedi et al. [73] presented a model which detected bacterial spots from images of peach leaves. Vallabhajosyula et al. [56] proposed a deep ensemble neural network to diagnose 38 classes of diseases from 14 plant species. Abbas et al. [28] suggested a methodology for identifying nine classes of diseases from images of tomato leaves, namely, yellow leaf curl virus, bacterial spot, septoria leaf spot, two-spotted spider mite, target spot, early blight, leaf mold, late blight, and mosaic virus. Chen et al. [71] suggested an approach which diagnosed 60 distinct classes of diseases from the leaf images of 26 plant species. 

Akshai et al. [31] proposed CNN-based models, which they utilized for diagnosing black rot, leaf blight, and esca from images of grape leaves, which were acquired from the plant village dataset. Malathy et al. [1] proposed a CNN for diagnosing diseases, namely, bitter rot, powdery mildew, and sooty blotch, from images of apples. Kibriya et al. [32] diagnosed early and late blight and bacterial spot, whereas Ashwinkumar et al. [66] proposed a model for identifying leaf mold, early and late blight, and target spot from images of tomato leaves. 

Table 12 shows a summarized view of the plant species and classes of diseases detected and classified by the reviewed studies.

#### Observation 9

**Figure 13:**
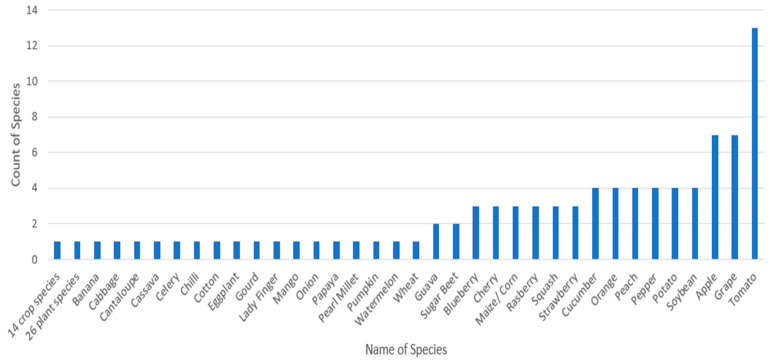
Species for which diagnosis was performed.

This observation is purely framed on the basis of the discussion for RQ9 (3.9). As per Figure 13, it is evident that the evaluated studies mostly worked on classifying tomato diseases (13 evaluated studies). Secondly, the number of evaluated studies classifying diseases for the apple and grape species was equal, at seven. Four evaluated studies were conducted to classify diseases in cucumber, orange, peach, pepper, potato, and soybean, whereas two reviewed studies included guava and sugar beet. Lastly, diseases in chili, papaya, cotton, wheat, pearl millet, etc., were diagnosed by one reviewed study. Figure 13 depicts the various species for which diagnoses were made by the evaluated studies.

### 3.10. Discussion for RQ10: What Is the Accuracy of Existing Plant Disease Detection and Classification Approaches?

This section focuses on accuracy of the existing approaches that were proposed by the evaluated studies.

Rumpf et al. [2], for the diagnosis of several diseases in sugar beet, proposed an SVM model based on hyperspectral reflectance which offered accuracy levels greater than 86%. Wang et al. [35] used a model for predicting two different grape diseases with an accuracy of 97.14%, while two types of wheat diseases were detected with 100% accuracy using BPNN and image processing technologies. Dubey et al. [3] proposed an approach that attained 93% accuracy in identifying various diseases in apples, namely, apple rot, apple scab, and apple blotch. Mahlein et al. [72] proposed a model for detecting sugar beet diseases which achieved accuracy rates for sugar beet rust, powdery mildew, and Cercospora leaf spot of 87%, 85%, and 92%, respectively. Sannakki et al. [37] proposed a model which achieved 100% accuracy in identifying two different grape illnesses by utilizing the hue feature. Es-saddy et al. [38] proposed a model that attained an accuracy of 87.80%. The proposed CNN model of Fujita et al. [9] attained 82.3% accuracy for detecting various cucumber diseases. Durmus et al. [41] used AlexNet and SqueezeNet, two DL-based models, which attained 95.65 and 94.3 percent accuracy, respectively, whereas a 99.18% accuracy rate was attained by Brahimi et al. [10], who used CNN for identifying the same illnesses from tomato leaves. 

Liu et al. [42] used an AlexNet-based model which attained 97.62% accuracy for identifying different apple diseases. Ferentinos [61] proposed a CNN model that obtained an accuracy of 99.53% for detecting 58 classes of diseases. Ramesh et al. [4] proposed a random forest classifier that provided an accuracy of 70% for detecting healthy and unhealthy papaya leaves. Ma et al. [13] attained an accuracy of 93.4% using the proposed deep CNN to identify various kinds of cucumber leaf diseases. Sardogan et al. [14] achieved 86% accuracy in the detection of septoria spot, bacterial spot, yellow curved, and late blight from tomato leaves by utilizing CNN and learning vector quantization. Behera et al. [5] proposed SVM with k-means clustering obtained an accuracy of 90% for detecting orange diseases. Geetharamani et al. [16] attained an accuracy of 96.46% for identifying diseases from the leaves of 13 different species of plants. Wahab et al. [7] proposed model provided an accuracy of 57.1% for detecting cucumber mosaic diseases from chili leaves. Francis et al. [62] suggested a CNN model which achieved 87% accuracy in the identification of diseases in apple and tomato leaf species. Ji et al. [20] proposed a UnitedModel, which attained a test accuracy of 98.57%. 

The proposed approach of Kumari et al. [70] attained 90% accuracy in identifying bacterial leaf spots and 80% in diagnosing target spots from cotton leaves, whereas it provided an accuracy of 100% in identifying two distinct classes of tomato diseases from its leaves. Zhang et al. [17] proposed a model which attained 94.65% accuracy for detecting downy mildew, anthracnose, black spot, powdery mildew, angular leaf spot, and gray mold diseases using a convolutional neural network with global average pooling from cucumber leaves. Haque et al. [46] proposed a CNN which achieved an accuracy of 95.61% for diagnosing diseases from guava, whereas the suggested deep CNN model of Howlader et al. [64] attained an accuracy of 98.74% in identifying fruit rot, anthracnose, and fruit canker from guava leaves. Sahithya et al. [47] proposed SVM and ANN models for identifying various diseases from lady finger leaves, which showed variation when tested using datasets with and without noise images. SVM provided an accuracy of 85% when noise was present in the images and 92% when tested for images without noise, whereas ANN provided 97% accuracy when noise was present in the images and 98% in images without noise. Coulibaly et al. [19] attained an accuracy of 95% in diagnosing mildew from pearl millet. Jadhav et al. [24] proposed an AlexNet which attained 98.75% accuracy, whereas GoogleNet attained 96.25% accuracy for identifying diseases from soybean leaves. Kannan E et al. [68] proposed a CNN model that obtained an accuracy of 97% in detecting diseases, namely, early blight, mosaic virus, septoria leaf spot, yellow leaf curl, and bacterial spot, from tomato leaves.

Sun et al. [26] proposed a discount momentum deep learning optimizer which attained an accuracy of 97% for detecting 26 classes of diseases. Pham et al. [51] suggested a model which achieved a testing accuracy of 85.45% for identifying diseases from mango leaves. Shrestha et al. [22] obtained an accuracy of 88.8% using the proposed CNN model for diagnosing diseases in tomato, potato, and bell pepper leaves. The research of Sujatha et al. [27] revealed that when it comes to identifying citrus plants, DL models showed a superior performance to ML models. Different ML models, such as SVM, stochastic gradient descent, and random forest, achieved accuracies of 87%, 86.5%, and 76.8%, respectively. In contrast, three DL models, namely, Inception-v3, VGG-16, and VGG-19, provided disease detection accuracies of 89%, 89.5%, and 87.4%, respectively, for the same species. Bedi et al. [73] suggested an approach based on CNN and convolutional autoencoders which attained an accuracy of 98.38%. Vallabhajosyula et al. [56] proposed a deep ensemble neural network technique which obtained 99.99% accuracy when the performance was assessed using the PlantVillage dataset. The accuracies of the C-GAN model, provided by Abbas et al. [28], for five classes, seven classes, and ten classes of tomato leaf images were 99.51%, 98.65%, and 97.11%, respectively. 

Chen et al. [71] suggested a model which attained an accuracy of 93.9% for identifying 60 classes of diseases from 26 different plant species. Akshai et al. [31] proposed a DenseNet model which achieved 98.27% accuracy for diagnosing black rot, leaf blight, and esca from images of grape leaves. Kibriya et al. [32] proposed two models for identifying diseases from tomato leaves, namely, GoogleNet and VGG16. GoogleNet obtained an accuracy of 99.23%, whereas VGG-16 attained 98% accuracy. Malathy et al. [1] proposed a CNN which obtained an accuracy of 97% for diagnosing diseases from images of apples. Ashwinkumar et al. [66] suggested an optimal mobile network-based CNN, which achieved an accuracy of 98.7% for detecting various diseases, namely, late blight, target spot, leaf mold, and early blight, from images of tomato leaves.

#### Observation 10

**Figure 14:**
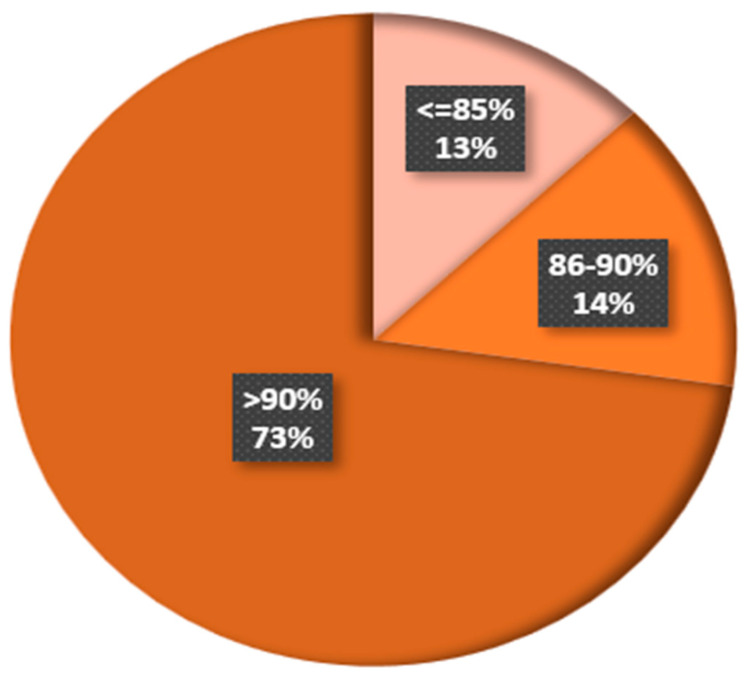
Classification accuracies of the evaluated studies.

This observation is solely based on the discussion of RQ10. Three categories were used to classify the accuracy levels attained by the various reviewed studies. The accuracies achieved by various existing models are compared in Figure 14 in three classes: ≤85%, 86–90%, and >90%. It was found that 73% of the evaluated studies offered plant disease diagnosis accuracy of more than 90%, while 14% offered accuracies of between 86 and 90%. The percentage of the examined studies with accuracy levels of 85% or less was only 13%.

## 4. Challenges in Existing Approaches

These discussions were solely based on the literature that was reviewed for plant diseases; the conclusions might be different for applications of image processing, ML, and DL in other fields.

- The analysis of disease classification can be impacted by environmental factors such as temperature and humidity;
- It is difficult to identify appropriate and unhealthy portions of leaves because disease symptoms are not well defined;
- Some models were unable to identify a certain stage of a plant leaf disease;
- Some models failed to extract the desired impacted area from images with intricate backgrounds;
- Several of the methods discussed in this review study were trained using the publicly available PlantVillage dataset, but they fell short when put to the test against a real-world environment.

## 5. Overall Observation and Comparison

**Figure 15:**
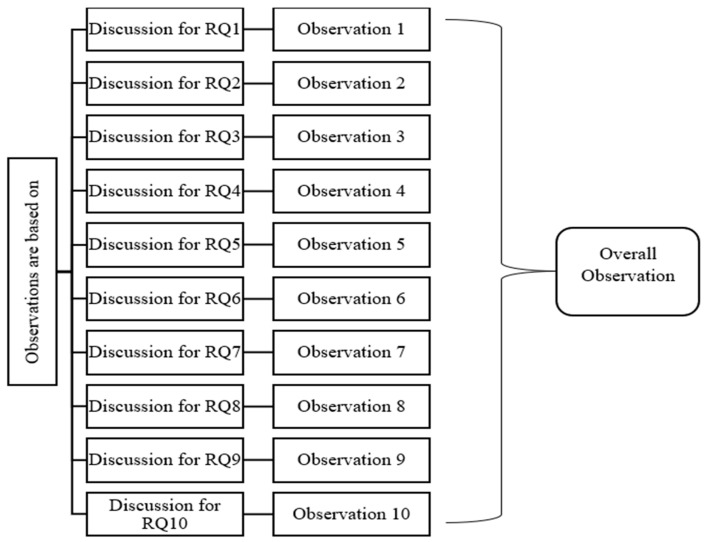
Flowchart showing how the observations were framed.

**Figure 16:**
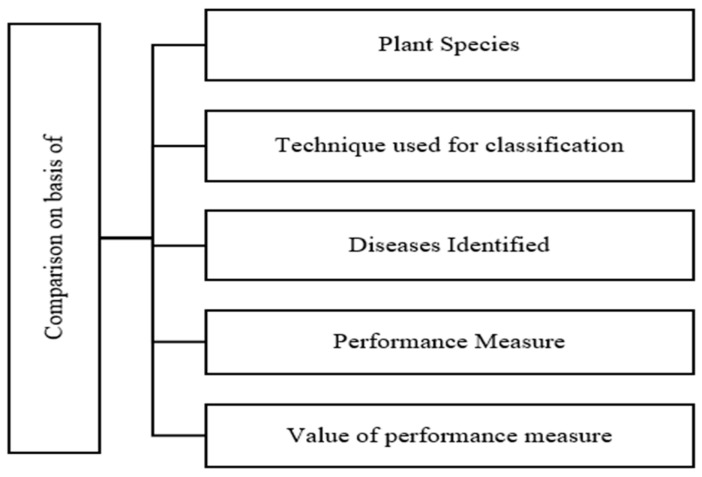
Flowchart showing how the comparison was framed.

This section involves overall observation and comparison. The overall observation was framed on the basis of Observations 1 to 10, as shown in Figure 15. The comparison section involves a comparison of various parameters, as shown in Figure 16.

### 5.1. Overall Observation

The majority of the reviewed studies obtained image data from publicly available datasets, as is evident from Observation 1. Secondly, Observation 2 indicates that resizing was utilized for pre-processing the images, whereas Observation 3 reflects the size of the dataset, or, simply, that the count of images in the dataset was increased using rotation operation during the data augmentation stage in most of the evaluated studies. Thirdly, Observation 4 indicates that during feature extraction, GLCM was widely utilized, and Observation 5 reflects the texture features extracted by most of the evaluated studies. The plant diseases were classified using CNN in many of the publications that were reviewed, as demonstrated by Observation 6. In the majority of the studies that were analyzed, the quality of the images was improved during pre-processing, as shown by Observation 7, while Observation 8 reveals that data augmentation helped to decrease the overfitting of the models. Last but not least, Observation 10 demonstrates that the majority of the reviewed studies offered accuracy levels greater than or equal to 90%.

### 5.2. Comparison

Table 13 involves a comparison of various reviewed papers on the basis of the species evaluated, the techniques used for identification, the disease identified, the performance measures, and their value.

## 6. Conclusions and Future Scope

Diverse available techniques using ML, DL, and image processing were surveyed in this research to determine their applicability to diagnosing illnesses in various plant species. By looking into the field of agriculture for this effort, 75 pertinent articles were selected for this review. Attention was particularly paid to the data sources, pre-processing methods, feature extraction methods, data augmentation methods, utilized models, and general effectiveness of proposed models. The results showed that most existing models have a modest capacity to process original image data in its unstructured state. For the purpose of separating the desired impacted area from the complicated background of an image, identification techniques based on different approaches required systematic engineering and expert design abilities.

This survey’s objective was to encourage researchers to use various image processing, ML, and DL approaches for identifying and categorizing plant diseases. Most of the reviewed studies worked on images of single leaves for disease detection; in future work, multiple leaves in a single frame may be used for disease detection. These images could be captured in diversified environmental conditions (temperature, humidity, etc.), for the purpose of reducing the impact of environmental conditions on disease detection, and new approaches could be developed which provide detail regarding the stage of the disease. Moreover, in the future, plant disease detection approaches can be integrated with drones and mobile applications to detect diseases in their early stages in large agricultural fields. 

## Figures and Tables

**Table 1:** Number of papers extracted utilizing each keyword.

List of Searched Keywords	Paper Extracted
Image processing	29
Deep learning	34
Plant disease classification	49
Machine learning	26
Convolutional neural network	27
Computer vision	17
Total	182

**Table 2:** Research questions and their motives.

S. No.	Research Questions	Motivation
1.	RQ1: What are the main sources for collecting data about plants?	To identify different data acquisition sources that are utilized by different researchers to collect plant image data.
2.	RQ2: What different pre-processing techniques are applied?	To identify different pre-processing techniques.
3.	RQ3: What different techniques are used for data augmentation?	To identify different data augmentation techniques that are utilized for increasing the size of the dataset.
4.	RQ4: What kinds of feature extraction methods are employed?	To identify different feature extraction techniques that are utilized for extracting features.
5.	RQ5: What are the typical attributes that are used or extracted?	To identify different extracted features.
6.	RQ6: What automated systems have been implemented for identifying and categorizing plant diseases?	To identify models that are implemented for identifying and categorizing plant diseases.
7.	RQ7: What analytical techniques are employed for improving image quality?	To identify techniques utilized for improving the quality of images.
8.	RQ8: What are the techniques utilized for reducing/removing overfitting?	To identify techniques used for reducing overfitting.
9.	RQ9: What are the different plant species on which the evaluated research is based, and what classifications of diseases have been found by the evaluated studies?	To identify the species with which evaluated studies are dealing and classes of diseases identified by reviewed studies.
10.	RQ10: What is the accuracy of existing plant disease detection and classification approaches?	To identify the accuracy of existing approaches for identifying plant diseases.

**Table 3:** Summarized view of data acquisition source.

S. No.	Year and Reference	Data Acquisition Source
1.	2010 [2]	Real Environment
2.	2012 [35]	Real Environment
3.	2012 [36]	Real Environment
4.	2013 [37]	Internet, Real Environment
5.	2016 [38]	Internet, Real Environment
6.	2016 [8]	Internet
7.	2016 [9]	Real Environment
8.	2016 [40]	PlantVillage dataset
9.	2017 [41]	PlantVillage dataset
10.	2017 [10]	PlantVillage dataset
11.	2017 [11]	Real Environment
12.	2017 [42]	Real Environment
13.	2018 [13]	Public Dataset, Real Environment
14.	2018 [14]	PlantVillage dataset
15.	2018 [12]	Real Environment
16.	2019 [16]	PlantVillage dataset
17.	2021 [43]	PlantVillage dataset
18.	2019 [44]	PlantVillage dataset
19.	2019 [7]	Real Environment
20.	2019 [45]	PlantVillage dataset
21.	2019 [46]	Real Environment, Interenet
22.	2019 [47]	Real Environment
23.	2020 [48]	Real Environment
24.	2020 [21]	Real Environment
25.	2020 [49]	Real Environment
26.	2020 [50]	Internet, Real Environment
27.	2020 [23]	Real Environment
28.	2021 [24]	Real Environment
29.	2021 [26]	PlantVillage dataset
30.	2021 [25]	PlantVillage dataset
31.	2021 [52]	PlantDoc Dataset
32.	2021 [28]	PlantVillage dataset
33.	2021 [53]	PlantVillage dataset
34.	2021 [29]	Public Dataset
35.	2021 [30]	PlantVillage dataset
36.	2021 [54]	PlantVillage dataset
37.	2021 [31]	PlantVillage dataset
38.	2021 [32]	PlantVillage dataset
39.	2021 [33]	PlantVillage dataset
40.	2021 [55]	Public Dataset
41.	2021 [27]	Real Environment
42.	2022 [34]	Public Dataset
43.	2022 [56]	PlantVillage dataset

**Table 4:** Real Environment description.

S. No.	Year and Reference	Real Environment Description
1.	2010 [2]	A Germany-based commercial substrate was used to grow sugar beets in order to perform experiments with sugar beet leaves in a greenhouse. Spectral reflectance was measured using a portable non-imaging spectroradiometer, and the SPAD-502 chlorophyll meter was used to determine the amount of chlorophyll.
2.	2012 [35]	Digital camera
3.	2012 [36]	Digital camera
4.	2013 [37]	Under the guidance of an expert, images of grape leaves were shot in Sangali, Pune, and Bijapur using a 16.1 Megapixel Nikon Coolpix P510 digital camera.
5.	2016 [38]	Under the supervision of an agricultural expert, images of leaves were captured from various farms with a digital camera.
6.	2016 [9]	Images of cucumber leaves taken with a digital camera were provided by Japan’s Research Center.
7.	2017 [11]	Camera devices were used to capture images of tomato leaves, stems, and fruits in Korea’s different farms at the early, medium, and late phases of disease.
8.	2017 [42]	Apple leaf images were taken from China (Baishui and Qingyang)
9.	2018 [13]	Images of cucumber leaves, with 2592 × 1944 resolution, were captured using a Nikon Coolpix S3100 from a greenhouse in Tianjin (China).
10.	2018 [12]	Images of cucumber leaves were provided by Japan’s Research Center.
11.	2019 [7]	Using the Raspberry Pi Camera V2, pictures of chili stalks were taken at various heights and angles.
12.	2019 [46]	The Nikon D7200 DSLR was used to take images of guava from several locations in different situations.
13.	2019 [47]	Lady finger leaf images of were photographed using a 1584 × 3456 resolution digital camera.
14.	2020 [48]	China’s Fujian Institute of Subtropical Botany supplied about 1000 leaf images of maize and rice. The shots were taken in environments with uneven lighting levels and cluttered field backgrounds.
15.	2020 [21]	Using a Samsung Intelligent LCD camera, images of healthy and diseased leaves were taken.
16.	2020 [49]	After several visits to farming regions, images of tomato leaves were gathered.
17.	2020 [50]	Images of rice and maize leaves were captured from research farms related to the China’s Fujian Institute Agricultural research farms.
18.	2020 [23]	The 8 MP Samsung A7 smartphone camera was used to take images of lady finger leaves from fields in two villages in the Tiruvannamalai region.
19.	2021 [24]	Images of soybean leaves were taken from soybean fields in India’s Kolhapur region.
20.	2021 [27]	Images of citrus leaves were captured with a 72 dpi resolution DSLR from a citrus research center in Sargodha City.

**Table 5:** Summarized view of various pre-processing techniques.

S. No.	Year and Reference	Operation Performed
1.	2013 [37]	anisotropic diffusion
2.	2015 [57]	image smoothing, clipping, histogram equalization, image enhancement, and color conversion
3.	2015 [58]	resizing and cropping
4.	2016 [38]	resizing, denoising
5.	2016 [8]	resizing and cropping
6.	2017 [59]	clipping, smoothing, image enhancement
7.	2018 [61]	size reduction and cropping
8.	2018 [5]	image enhancement, CIELAB color space
9.	2019 [62]	resizing and cropping
10.	2019 [63]	downsize images, improve contrast, and transform RGB images into greyscale
11.	2019 [7]	RGB format images into grayscale
12.	2019 [18]	enhancing compactness, changing brightness, extracting noise, and converting to another color space
13.	2019 [47]	resizing operation
14.	2020 [51]	downscaled to a lower resolution, contrast enhancement
15.	2021 [30]	resizing and normalization
16.	2021 [32]	resizing and denoising
17.	2021 [65]	resizing, restoration, and image enhancement
18.	2021 [1]	image resizing and image restoration
19.	2022 [66]	denoising

**Table 6:** Summarized view of different data augmentation techniques.

S. No.	Year and Reference	Augmentation Operation Performed
1.	2016 [8]	Rotations, 3 × 3 transformation matrix-based perspective transformation, Affine transformations
2.	2016 [9]	Image shifting, image rotation, and image mirroring
3.	2016 [39]	Rotation and mirroring
4.	2017 [11]	Geometrical transformation and intensity transformations
5.	2018 [13]	Rotation and flipping operations
6.	2018 [12]	Cropping and rotation
7.	2018 [67]	Rotation, shear conversion, cutout, and horizontal and vertical direction movement
8.	2019 [16]	Flipping, principal component analysis (PCA), color augmentation, rotation, scaling, noise injection, and gamma correction
9.	2019 [17]	Intensity transformations and geometric transformations
10.	2019 [15]	RandomRotate, RandomFlip, and RandomLighting
11.	2019 [45]	Cropping, flipping, shifting, rotating
12.	2019 [46]	Flipping (horizontal flip), zooming, shifting (height and breadth), rotating, nearest fill, and shearing
13.	2019 [19]	Rescale, flipping, shift, and zoom
14.	2020 [20]	Rotation, zooming, flipping, shearing, and color changing
15.	2020 [48]	Rotation, flip, scaling, and translation
16.	2020 [68]	RandomResizedCrop, RandomRotation
17.	2020 [21]	Flip, rotation, and shift
18.	2020 [23]	Rotation, flipping (horizontally), shear, zoom, and shift (height, width)
19.	2021 [25]	Rotation, contrast enhancement, brightness enhancement, and noise reduction
20.	2021 [29]	SMOTE
21.	2021 [30]	Affine transformation
22.	2021 [31]	Rotation, shifting, and zooming
23.	2021 [54]	Horizontal flipping and four-angle rotation
24.	2021 [33]	Flip transformation
25.	2021 [69]	Rotation, filling, flipping, zooming, and shearing
26.	2022 [34]	Neural style transfer, position and color augmentation, deep convolutional generative adversarial network, and PCA
27.	2022 [56]	Scaling, translation, rotation, and image enhancement

**Table 7:** Summarized view of different feature extraction techniques.

S. No.	Year and Reference	Technique Utilized
1.	2012 [3]	Global color histogram, color coherence vector, local binary pattern, complete local binary pattern methods
2.	2013 [37]	Color co-occurrence
3.	2015 [58]	GLCM
4.	2016 [38]	GLCM, color histogram, color structure descriptor, and color moments
5.	2017 [59]	Color co-occurrence approach
6.	2017 [60]	GLCM
7.	2018 [4]	Histogram of oriented gradients
8.	2018 [5]	GLCM
9.	2019 [63]	GLCM
10.	2019 [6]	GLCM
11.	2019 [7]	GLCM
12.	2019 [47]	GLCM
13.	2021 [71]	RESNET18 (CNN) and a task-adaptive procedure
14.	2021 [65]	Scale-invariant feature transform
15.	2021 [55]	GLCM

**Table 8:** Summary of different extracted features.

S. No.	Year and Reference	Extracted Features
1.	2012 [35]	Color features (21), texture features (25), shape features (4)
2.	2012 [3]	Color feature, texture feature
3.	2013 [37]	Texture feature
4.	2015 [58]	Correlation, homogeneity, energy, and contrast
5.	2016 [38]	Color features, shape features, texture features
6.	2017 [59]	Texture features (cluster shade, energy, local homogeneity, contrast, and cluster prominence), color features
7.	2017 [60]	Texture features
8.	2018 [4]	Feature vectors
9.	2018 [5]	Texture features (mean, entropy, variance, kurtosis, smoothness, skewness, inverse difference moment (IDM), contrast, energy, correlation, homogeneity, standard deviation, and RMS)
10.	2019 [6]	Shape and texture features
11.	2019 [70]	Energy, correlation, variance, mean, contrast, standard deviation, homogeneity
12.	2019 [7]	Shape feature (4), texture feature (4)
13.	2019 [47]	Color features, geometrical features, texture features
14.	2021 [55]	Color features, texture features

**Table 9:** Summary of different classification techniques.

S. No.	Year and Reference	Classification Technique Used
1.	2010 [2]	SVM
2.	2012 [35]	Backpropagation networks
3.	2012 [3]	Multi-class SVM
4.	2013 [72]	Spectral disease indices
5.	2013 [37]	Feed-forward back propagation neural network
6.	2016 [38]	Two support vector machines (serial combination)
7.	2016 [9]	CNN
8.	2017 [41]	SqueezeNet, AlexNet
9.	2017 [10]	CNN
10.	2017 [42]	AlexNet
11.	2018 [61]	CNN models
12.	2018 [4]	Random forest
13.	2018 [13]	Deep CNN
14.	2018 [14]	CNN model based on LVQ
15.	2018 [5]	SVM
16.	2019 [16]	Deep CNN
17.	2019 [62]	CNN
18.	2019 [17]	Convolutional neural network with global average pooling
19.	2019 [7]	SVM
20.	2019 [15]	NASNet
21.	2019 [64]	Deep CNN
22.	2019 [46]	CNN
23.	2019 [47]	ANN and SVM
24.	2020 [20]	CNN
25.	2021 [24]	AlexNet and GoogleNet
26.	2021 [26]	DM deep learning optimizer
27.	2020 [22]	CNN
28.	2021 [73]	CNN and convolutional autoencoders
29.	2021 [28]	DenseNet
30.	2021 [31]	VGG, DenseNet, and ResNet
31.	2021 [32]	GoogleNet, VGG16
32.	2021 [27]	SVM, stochastic gradient descent, and random forest (machine learning) Inception-v3, VGG-16, and VGG-19 (deep learning)
33.	2022 [66]	Optimal mobile network-based CNN

**Table 10:** Summary of different techniques utilized for improving image quality.

S. No.	Year and Reference	Quality of Images Was Improved
1.	2012 [35]	Median filter
2.	2014 [74]	Histogram equalization and color conversion
3.	2015 [57]	Histogram equalization
4.	2016 [38]	During pre-processing
5.	2017 [59]	During pre-processing
6.	2019 [6]	During pre-processing
7.	2021 [1]	During pre-processing
8.	2022 [75]	LeafGAN
9.	2022 [56]	During pre-processing
10.	2022 [66]	Bilateral filter

**Table 11:** Summary of ways for reducing or removing overfitting.

S. No.	Year and Reference	Technique Utilized from Reducing Overfitting
1.	2016 [8]	Distortion was introduced
2.	2016 [9]	Rotation and flipping (data augmentation)
3.	2017 [41]	Dropout layers and pooling layers
4.	2017 [11]	Extensive data augmentation
5.	2017 [42]	Image processing, response-normalizing layers, swapping out some fully connected layers for convolution layers
6.	2018 [13]	Data augmentation
7.	2019 [16]	Distorted images were added
8.	2019 [62]	Dropout value—0.25
9.	2019 [64]	ReLU activation function, data augmentation approaches
10.	2019 [19]	Early stopping strategy
11.	2020 [48]	Data augmentation
12.	2020 [20]	Early stop mechanism, data augmentation techniques, and dropout
13.	2020 [68]	Data augmentation
14.	2021 [25]	Data augmentation
15.	2021 [28]	Data augmentation
16.	2021 [73]	Early halting
17.	2021 [53]	1 × 1 convolution
18.	2021 [71]	Forward propagation
19.	2021 [30]	Global average pooling
20.	2022 [34]	Data augmentation
21.	2022 [56]	Data augmentation

**Table 12:** Summary of plant species and classes of detected and classified diseases.

S. No.	Year and Reference	Plant Species	Detected Diseases
1.	2010 [2], 2013 [72]	Sugar beet	Sugar beet rust, powdery mildew, Cercospora leaf spot
2.	2012 [35]	Wheat, grape	Powdery mildew, downy mildew, stripe rust, leaf rust
3.	2012 [3]	Apple	Rot, blotch, scab
4.	2013 [37]	Grape	Downy mildew, powdery mildew
5.	2016 [38]	-	Pathogens (early blight, late blight, powdery mildew), pest insects (thrips, Tuta absoluta, leaf miners)
6.	2016 [9]	Cucumber	Mosaic viruses (zucchini yellow, cucumber mosaic virus, watermelon mosaic virus, and kyuri green mottle mosaic virus) and other viruses, including melon yellow spot virus, cucurbit chlorotic yellows virus, and papaya ring spot virus
7.	2017 [10], 2017 [41]	Tomato	Multiple
8.	2017 [42]	Apple	Rust, alternaria leaf spot, mosaic, and brown spot
9.	2020 [51]	Multiple	Multiple
10.	2021 [4]	Papaya	Unhealthy class
11.	2018 [13]	Cucumber	Powdery mildew, target leaf spots, downy mildew, anthracnose
12.	2018 [14]	Tomato	Bacterial spot, yellow curved, septoria spot, late blight
13.	2018 [5]	Orange	Citrus canker, brown rot, melanoses, stubbornness
14.	2019 [16]	Multiple	Multiple
15.	2019 [62]	Apple, tomato	Healthy/unhealthy
16.	2020 [20]	Grape	Esca, isariopsis leaf spot, black rot
17.	2019 [70]	Cotton, tomato	Cotton (target spot, bacterial leaf spot), tomato (septoria leaf spot, leaf mold)
18.	2019 [17]	Cucumber	Anthracnose, gray mold, powdery mildew, black spot, angular leaf spot, downy mildew
19.	2019 [7]	Chili	Cucumber mosaic virus
20.	2019 [46]	Guava	Anthracnose, fruit canker, fruit rot
21.	2019 [64]	Guava	Whitefly, algal leaf spot, rust
22.	2019 [47]	Lady finger	Leaf spot, yellow mosaic vein, powdery mildew
23.	2019 [19]	Pearl millet	Mildew
24.	2021 [24]	Soybean	Frogeye leaf spots, bacterial blight, brown spots
25.	2020 [68]	Tomato	Septoria leaf spot, yellow leaf curl, bacterial spot, early blight, mosaic virus
26.	2021 [26]	Multiple	Multiple
27.	2020 [51]	Mango	Gall midge, anthracnose, powdery mildew
28.	2020 [22]	Tomato, bell pepper, potato	Potato (early and late blight), pepper (bacterial spot), tomato (target spot, yellow leaf curl virus, mosaic virus, septoria leaf spot, early blight, spider mites, bacterial spot, leaf mold, late blight)
29.	2021 [73]	Peach	Bacterial spots
30.	2022 [56]	Multiple	Multiple
31.	2021 [28]	Tomato	Bacterial spot, yellow leaf curl virus, septoria leaf spot, two-spotted spider mite, early and late blight, target spot, leaf mold, mosaic virus
32.	2021 [71]	Multiple	Multiple
33.	2021 [31]	Grape	Leaf blight, black rot, esca
34.	2021 [1]	Apple	Bitter rot, sooty blotch, powdery mildew
35.	2021 [32]	Tomato	Late blight, bacterial spot, early blight
36.	2022 [66]	Tomato	Late blight, leaf mold, target spot, early blight
37.	2022 [76]	Multiple	Multiple
38.	2021 [77]	Multiple	Multiple
39.	2022 [78]	Multiple	Multiple
40.	2022 [79]	Multiple	Multiple

**Table 13:** Comparison of various reviewed papers.

S. No.	Year and Reference	Species	Techniques Used	Disease Identified	Performance Measure	Value
1.	2010 [2]	Sugar beet	SVM based on hyperspectral reflectance	Sugar beet rust, Cercospora leaf spot, powdery mildew	Accuracy	Higher than 86%
2.	2012 [35]	Grapes, Wheat	Backpropagation networks, image processing technologies	Grape (downy mildew, powdery mildew), wheat (stripe rust, leaf rust)	Accuracy (prediction accuracy, fitting accuracy)	Fitting accuracy—100% (for both), prediction accuracy—97.14% (grape), 100% (wheat)
3.	2012 [3]	Apple	Image processing techniques (multi-class SVM)	Apple rot, apple scab, apple blotch	Accuracy	93%
4.	2013 [72]	Sugar beet	Spectral disease indices	Sugar beet rust, cercospora leaf spot, powdery mildew	Accuracy	Sugar beet rust—87%, cercospora leaf spot—92%, powdery mildew—85%
5.	2013 [37]	Grape	Feed-forward back propagation neural network	Powdery mildew, downy mildew	Accuracy	100% (using the HUE feature only)
6.	2016 [38]	-	SVM (serial combination of two SVMs)	Thrips, Tuta absoluta, leaf miners (damaged by pest insects), early blight, powdery mildew, late blight (pathogens symptoms)	Accuracy	87.80%
7.	2016 [9]	Cucumber	Convolutional neural network	KGMMV, WMV, PRSV, CMV, CCYV, ZYMV, MYSV	Accuracy	82.3%
8.	2017 [41]	Tomato	Deep learning (AlexNet and SqueezeNet)	Spider mites, yellow leaf curl virus, early blight, bacterial spot, septoria leaf spot, leaf mold, late blight, mosaic virus, target spot	Accuracy	AlexNet—95.65%, SqueezeNet—94.3%
9.	2017 [10]	Tomato	CNN	Yellow leaf curl virus, bacterial spot, late blight, leaf mold, spider mites, septoria spot, mosaic virus, target spot, early blight	Accuracy	99.18%
10.	2017 [42]	Apple	Deep convolutional neural network (AlexNet)	Rust, mosaic, alternaria leaf spot, brown spot	Accuracy	97.62%
11.	2018 [61]	25 different plant species	CNN models based on deep learning techniques	58 distinct classes	Accuracy	99.53%
12.	2018 [4]	Papaya	Random forest (RF)	Healthy/unhealthy	Accuracy	70%
13.	2018 [13]	Cucumber	DCNN	Downy mildew, anthracnose, powdery mildew, and target leaf spots	Accuracy	93.4%
14.	2018 [14]	Tomato	CNN with learning vector quantization	Septoria spot, bacterial spot, yellow curved, and late blight	Accuracy	86%
15.	2018 [5]	Orange	SVM with K-means clustering (classification), degree of disease severity—fuzzy logic	Brown rot, citrus canker, melanoses, stubborn	Accuracy	90%
16.	2019 [16]	13 different plant leaves (grape, apple, tomato, cherry, peach, potato, and others)	Nine-layer deep CNN	Potato (early blight), cherry (powdery mildew), apple with black rot, peach with bacterial spots, tomato (leaf mold), grape (leaf blight), etc.	Accuracy	96.46%
17.	2019 [62]	Apple, tomato	Convolutional neural network	Healthy/diseased	Accuracy	87%
18.	2020 [20]	Grape	Convolutional neural network (UnitedModel)	Esca, black rot, isariopsis	Validation accuracy, test accuracy	test accuracy—98.57%, validation accuracy—99.17%
19.	2019 [70]	Cotton, tomato	Image processing techniques, neural network	Cotton (target spot, bacterial leaf spot), tomato (septoria leaf spot, leaf mold)	Accuracy	For cotton (bacterial leaf spot—90%, target spot)—80%, for tomato (septoria leaf spot and leaf mold)—100%
20.	2019 [17]	Cucumber	CNN with global average pooling	Black spot, powdery mildew, angular leaf spot, gray mold, anthracnose, downy mildew	Accuracy	94.65%
21.	2019 [7]	Chili	SVM	Cucumber mosaic virus	Accuracy	57.1%
22.	2019 [64]	Guava	Deep convolutional neural network	Rust, algal leaf spot, whitefly	Accuracy	98.74%
23.	2019 [46]	Guava	Convolutional neural network	Anthracnose, fruit canker, fruit rot	Accuracy	95.61%
24.	2019 [47]	Lady finger	SVM, artificial neural network	Powdery mildew, leaf spot, yellow mosaic vein	Accuracy	85% (SVM) and 97% (ANN); without noise, 92% (SVM) and 98% (ANN)
25.	2019 [19]	Pearl millet	Transfer learning with feature extraction	Mildew	Accuracy, f1-score, recall, precision	Accuracy—95%, f1-score—91.75%, recall—94.50%, precision—90.50%
26.	2021 [24]	Soybean	CNN (GoogleNet, AlexNet)	Brown spot, frogeye leaf spot, bacterial blight	Accuracy	98.75% (AlexNet), 96.25% (GoogleNet)
27.	2020 [68]	Tomato	Convolutional neural network	Septoria leaf spot, early blight, mosaic virus, yellow leaf curl virus, bacterial spot	Accuracy	97%
28.	2021 [26]	14 crops	Discount momentum deep learning optimizer	26 disease classes	Accuracy	97%
29.	2020 [51]	Mango	Feed-forward neural network (deep neural networks)	Powdery mildew, gall midge, anthracnose	Accuracy	91.32% (training accuracy), 85.45% (testing accuracy)
30.	2020 [22]	Potato, tomato, bell pepper	CNN	Potato (early and late blight), bell pepper bacterial spot, tomato (target spot, mosaic virus, early blight, bacterial spot, yellow leaf curl virus, late blight, septoria leaf spot, spider mites, leaf mold)	Test Accuracy	88.8%
31.	2021 [73]	Peach	Hybrid approach (convolutional autoencoder, convolutional neural network)	Bacterial spot	Accuracy	Testing accuracy—98.38%, training accuracy—99.35%
32.	2022 [56]	14 crops	Deep ensemble neural network	38 classes	Accuracy	99.99%
33.	2021 [28]	Tomato	C-GAN (for producing synthetic images), DenseNet	Two-spotted spider mite, bacterial spot, septoria leaf spot, yellow leaf curl virus, target spot, early blight, leaf mold, late blight, mosaic virus	Accuracy	99.51% (5 classes), 98.65% (7 classes), 97.11% (10 classes)
34.	2021 [71]	26 plant species	LFM-CNAPS based on meta-learning	60 diseases	Accuracy	93.9%
35.	2021 [31]	Grape	CNN (VGG, DenseNet, ResNet)	Black rot, leaf blight, esca	Accuracy	98.27% (DenseNet accuracy)
36.	2021 [32]	Tomato	GoogleNet, VGG16	Bacterial spot, early blight, late blight	Accuracy	GoogleNet—99.23%, VGG16—98%
37.	2021 [1]	Apple	Convolutional neural networks	Bitter rot, powdery mildew, sooty blotch	Accuracy	97%
38.	2022 [66]	Tomato	Optimal mobile network-Based CNN	Late blight, target spot, leaf mold, and early blight	Accuracy, recall, precision, kappa, F-score	98.7% (accuracy), 0.9892 (recall), 0.985 (precision, F1-score, kappa)

## Data Availability

Data will be available on request.
